# Identification of novel hub genes associated with gastric cancer using integrated bioinformatics analysis

**DOI:** 10.1186/s12885-021-08358-7

**Published:** 2021-06-14

**Authors:** Xiao-Qing Lu, Jia-Qian Zhang, Sheng-Xiao Zhang, Jun Qiao, Meng-Ting Qiu, Xiang-Rong Liu, Xiao-Xia Chen, Chong Gao, Huan-Hu Zhang

**Affiliations:** 1 Department of Breast Surgery, Shanxi Cancer Hospital, Taiyuan, Shanxi China; 2 Department of Rheumatology, the Second Hospital of Shanxi Medical University, Taiyuan, Shanxi China; 3 Department of Pathology, Brigham and Women’s Hospital, Harvard Medical School, Boston, MA USA; 4 Department of Gastroenterology, Shanxi Cancer Hospital, Taiyuan, 030001 Shanxi China

**Keywords:** Gastric cancer, Bioinformatics analysis, Differentially expressed genes

## Abstract

**Background:**

Gastric cancer (GC) is one of the most common solid malignant tumors worldwide with a high-recurrence-rate. Identifying the molecular signatures and specific biomarkers of GC might provide novel clues for GC prognosis and targeted therapy.

**Methods:**

Gene expression profiles were obtained from the ArrayExpress and Gene Expression Omnibus database. Differentially expressed genes (DEGs) were picked out by R software. The hub genes were screened by cytohubba plugin. Their prognostic values were assessed by Kaplan–Meier survival analyses and the gene expression profiling interactive analysis (GEPIA). Finally, qRT-PCR in GC tissue samples was established to validate these DEGs.

**Results:**

Total of 295 DEGs were identified between GC and their corresponding normal adjacent tissue samples in E-MTAB-1440, GSE79973, GSE19826, GSE13911, GSE27342, GSE33335 and GSE56807 datasets, including 117 up-regulated and 178 down-regulated genes. Among them, 7 vital upregulated genes (HMMR, SPP1, FN1, CCNB1, CXCL8, MAD2L1 and CCNA2) were selected. Most of them had a significantly worse prognosis except SPP1. Using qRT-PCR, we validated that their transcriptions in our GC tumor tissue were upregulated except SPP1 and FN1, which correlated with tumor relapse and predicts poorer prognosis in GC patients.

**Conclusions:**

We have identified 5 upregulated DEGs (HMMR, CCNB1, CXCL8, MAD2L1, and CCNA2) in GC patients with poor prognosis using integrated bioinformatical methods, which could be potential biomarkers and therapeutic targets for GC treatment.

**Supplementary Information:**

The online version contains supplementary material available at 10.1186/s12885-021-08358-7.

## Background

Gastric cancer (GC), the fifth most frequently diagnosed cancer and the third leading cause of cancer-related death [1], has become a major global health challenge. About 934,000 new GC cases and 700,000 mortalities occurred annually [2]. Despite improvement in diagnosis and treatment, the prognosis of GC patients remains poor, which has become an active topic of clinical and basic research. Genetic mutations, epigenetic alterations and aberrant molecular signaling pathways are involved in the processes of gastric carcinogenesis, spread and metastasis [3]. In particular, the new molecular characteristics can be applied in early risk assessment, the identification of better specific biomarkers, and the improvement of clinic treatment and survival.

In recent decades, microarray and high-throughput sequencing have been considered as reliable techniques to quickly detect differentially expressed genes (DEGs) [4] that are able to make various slice data be produced and stored in public databases. Consequently, many valuable clues could be explored for new research on the base of these data. However, with the data getting updated, a large amount of genetic information uploaded to public databases was not used effectively.

In this study, we downloaded related mRNA expression datasets from ArrayExpress and Gene Expression Omnibus. A set of DEGs in these datasets were extracted by comparing gene expression profiles of carcinoma specimen and adjacent normal tissues. By analyzing the GO and Kyoto Encyclopedia of Gene and Genome (KEGG) pathway enrichment [5, 6], along with the construction of protein–protein interaction (PPI) network [7], we selected vital genes. After evaluating the clinical prognosis of these genes and their transcriptional factor (TF) regulatory network, we further validated these genes by quantitative real-time PCR (qRT-PCR) in GC tissue samples.

## Methods

### Gastric cancer microarray data information

**Fig. 1:**
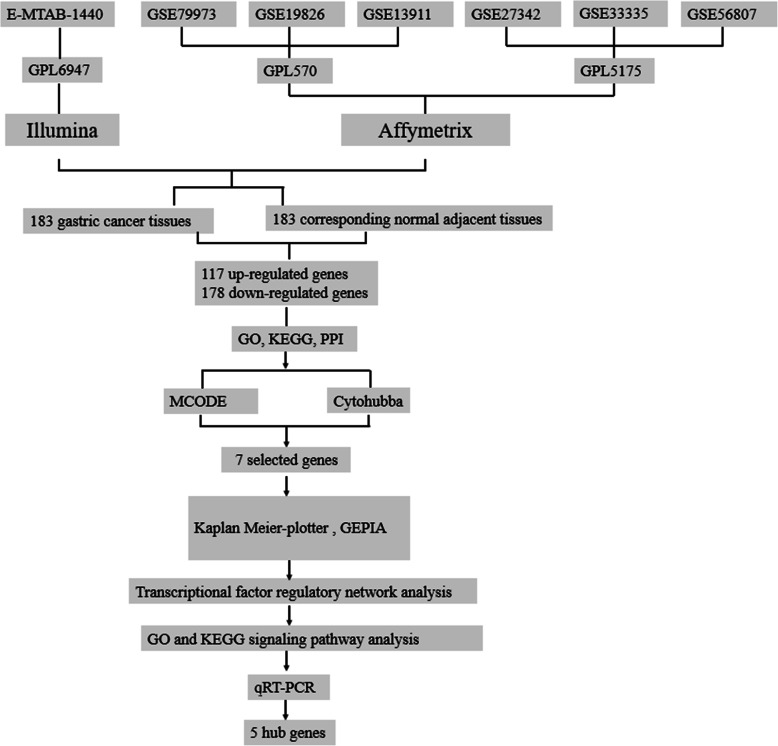
Flowchart of the multistep screening strategy used in this study on bioinformatics data in Arrayexpress and GEO database

Microarray data information of GC and adjacent gastric tissues were obtained from Arrayexpress (https://www.ebi.ac.uk/arrayexpress/) and NCBI-GEO (https://www.ncbi.nlm.nih.gov/geo). When “gastric cancer” was used as a keyword to perform queries, we selected the original studies of RNA assay and array assay in *Homo sapiens* which samples with available clinical information for analysis. The expression microarray datasets E-MTAB-1440, GSE79973, GSE19826, GSE13911, GSE27342, GSE33335 and GSE56807 were downloaded. Overall, 183 patients with gastric cancer enrolled in this study. The workflow chart is shown in Fig.1.

### Gene expression profile data

Microarray data of 7 databases were on account of three platforms. E-MTAB-1440 genome-wide gene expression profile data were generated from the Illumina platform GPL6947 (A-MEXP-1171-Illumina Human HT-12 v3.0 Expression BeadChip). GSE19826, GSE13911 and GSE27342 microarray data from the Affymetrix platform GPL570 (HG-U133_Plus_2 Affymetrix Human Genome U133 Plus 2.0 Arrays) and GSE27342, GSE33335 and GSE56807 microarray data from the Affymetrix platform GPL5175(HuEx-1_0-st-v1 Affymetrix Gene Chip Human Exon 1.0 ST Array version 1). Detailly, GPL6947 dataset consisted of 20 GC tissues and 20 adjacent normal gastric samples. GPL570 and GPL5175 respectively include 53 and 110 GC tissues as well as same number of matched normal specimen.

### Data processing of DEGs

Significant DEGs between GC specimen and normal gastric tissues specimen were analyzed via software and packages from Bioconductor (http://www.bioconductor.org/) in R (version 3.6.0). The microarray data were first preprocessed using the RMA (robust multi-array average) which contains background adjustment, normalization with the quantile method, and expression calculations. The probes were removed when they were not able to be matched to a specific gene symbol, and the average value was taken as the expression value for each gene when different probes matched to the same gene symbol. Then the statistically significant DEGs was selected by Moderated T statistic approach with “limma [8]” and “oligo [9]” package of Bioconductor. After preprocessing, SVA batch difference processing of combat was used to consolidate these 7 datasets to obtain the final dataset (GC tissues: corresponding normal adjacent tissues =183:183). Finally, DEGs were annotated through annotation table downloaded from the GEO website. The resulting *P* values were adjusted by the default Benjamini & Hochberg (BH) false discovery rate method. The adj. P value < 0.05, P value < 0.05 and |log fold change (FC)| > 0.58 were considered as significantly different for DEGs.

### Protein–protein interactions (PPI) network and module analysis

Information of DEGs’ protein experimental interactions and prediction was obtained by Search Tool for the Retrieval of Interacting Genes (STRING, Version 11.0, http://www.string-db.org/) [10] with the parameters set to species = H*omo sapiens*, and PPI score ≥ 0.4 (medium confidence) [11]. Subsequently, a specific PPI network of DEGs was constructed by cytoscape (version 3.7.2, http://www.cytoscape.org/) [12] based on the interactions retrieved from STRING. The gene-interaction relationship was represented by nodes and edges graphically for better visualization, which included phosphorylation, dephosphorylation, inhibition and activation. In the signaling network, the size of the cycle was considered as the frequency of the gene interaction. The most prominent central genes in the network indicated the genes with the highest frequency. In addition, the molecular complex detection (MCODE) analysis (Version 3.7.2, http://apps.cytoscape.org/apps/MCODE) [13] in cytoscape was used to identify the significant modules of the PPI network with degree cut-off 2, max depth 100, k-core 2, and node score cutoff 0.2. To screen the hub genes that may be involved in GC, we applied the cytohubba plug-in, using various parameters such as degree, betweenness centrality, and closeness. The DEGs from cytohubba were then subjected to VEEN analysis using the online tool (http://bioinformatics.psb.ugent.be/webtools/Venn/), and overlapping genes were considered selected genes.

### Evaluation of prognostic value of selected genes

Expression and prognostic values of the hub genes were analyzed using two online datasets, Kaplan Meier-plotter dataset (http://kmplot.com/analysis/) and Gene Expression Profiling Interactive Analysis (GEPIA, http://gepia.cancer-pku.cn) [14]. The hazard ratio (HR) with 95% confidence intervals and log rank *p* value were calculated and displayed on the plot. GEPIA was established for customized genomic analysis based on the Cancer Genome Atlas (TCGA) database, which was used to compare poor prognosis related hub genes expression between GC patients and healthy people.

### Transcriptional factor (TF) regulatory network construction

NetworkAnalyst (http://www.networkanalyst.ca/faces/home.xhtml) is used to explore TF-gene interactions for the input genes and assess the effect of the TF on the expression and functional pathways of the hub gene. In this study, the TFs of the hub genes were predicted from this database and a transcriptional regulatory network was constructed and visualized by the cytoscape software.

### Analysis of significant functions and pathway enrichment

After computing hub genes and evaluating prognosis, the database for annotation, visualization and integrated discovery (DAVID 6.8, http://david.abcc.ncifcrf.gov/) [6] was applied to re- analyze the KEGG pathway and Gene Ontology annotations for selected hub genes. *P*-value < 0.05, and count ≥2 were considered to indicate significance.

### Validation of selected DEGs’ transcription in fresh GC tissue specimens using quantitative real-time PCR

We analyzed samples from 10 GC patients who underwent tumor resection at the Department of Pathology, Shanxi Cancer Hospital (Shanxi, China). The detailed clinicopathological information for all the enrolled patients was available. GC and their corresponding normal adjacent tissue samples were immediately frozen in liquid nitrogen and stored at − 80 °C until further processing. Every specimen was anonymously handled based on ethical standards. All patients provided written informed consent and our study was approved by the hospital’s Ethical Review Committee.

The total RNA was extracted using Trizol reagent and reverse-transcribed into complementary DNA (cDNA) for quantitative real-time polymerase chain reaction (qRT-PCR) following the manufacturer’s instructions. GAPDH gene served as an endogenous control. The primer sequences of selected genes (HMMR, SPP1, FN1, CCNB1, CXCL8, MAD2L1 and CCNA2) used in the experiment are illustrated in Table 1. Each sample was tested in triplicates, and each sample underwent a melting curve analysis to check for the specificity of amplification. The relative expression level was determined as a ratio between the hub genes and the internal control GAPDH in the same mRNA sample, and calculated by the comparative CT method. Levels of hub genes’ expression were calculated by the 2^−ΔΔCt^ method [15, 16].

**Table 1:** Primer sequences of PCR

gene	Forward primer(5–3)	Reverse primer(5–3)
**HMMR**	GCTAAGCAAGAAGGCATGGA	CCACTTGATCTGAAGCACAAC
**SPP1**	GCCGAGGTGATAGTGTGGTT	AACGGGGATGGCCTTGTATG
**FN1**	AATAGATGCAACGATCAGGACA	GCAGGTTTCCTCGATTATCCTT
**CCNB1**	GACTTTGCTTTTGTGACTGACA	CCCAGACCAAAGTTTAAAGCTC
**CXCL8**	AACTGAGAGTGATTGAGAGTGG	ATGAATTCTCAGCCCTCTTCAA
**MAD2L1**	ACGGTGACATTTCTGCCACT	TGGTCCCGACTCTTCCCATT
**CCNA2**	AGAAACAGCCAGACATCACTAA	TTCAAACTTTGAGGCTAACAGC
**GAPDH**	ACAGTCAGCCGCATCTTCTT	ACGACCAAATCCGTTGACTC

### Statistical analysis

Demographic and clinical data were analyzed using Chi-squared test, student’s t-test or paired t-test to evaluate group balance of variables. All statistical analyses were performed using SPSS 26.0, the GraphPad Prism V8.0 and R 3.6.0. Two-tailed *P* < 0.05 were considered statistically significant.

## Results

### Identification of DEGs in GC

**Fig. 2:**
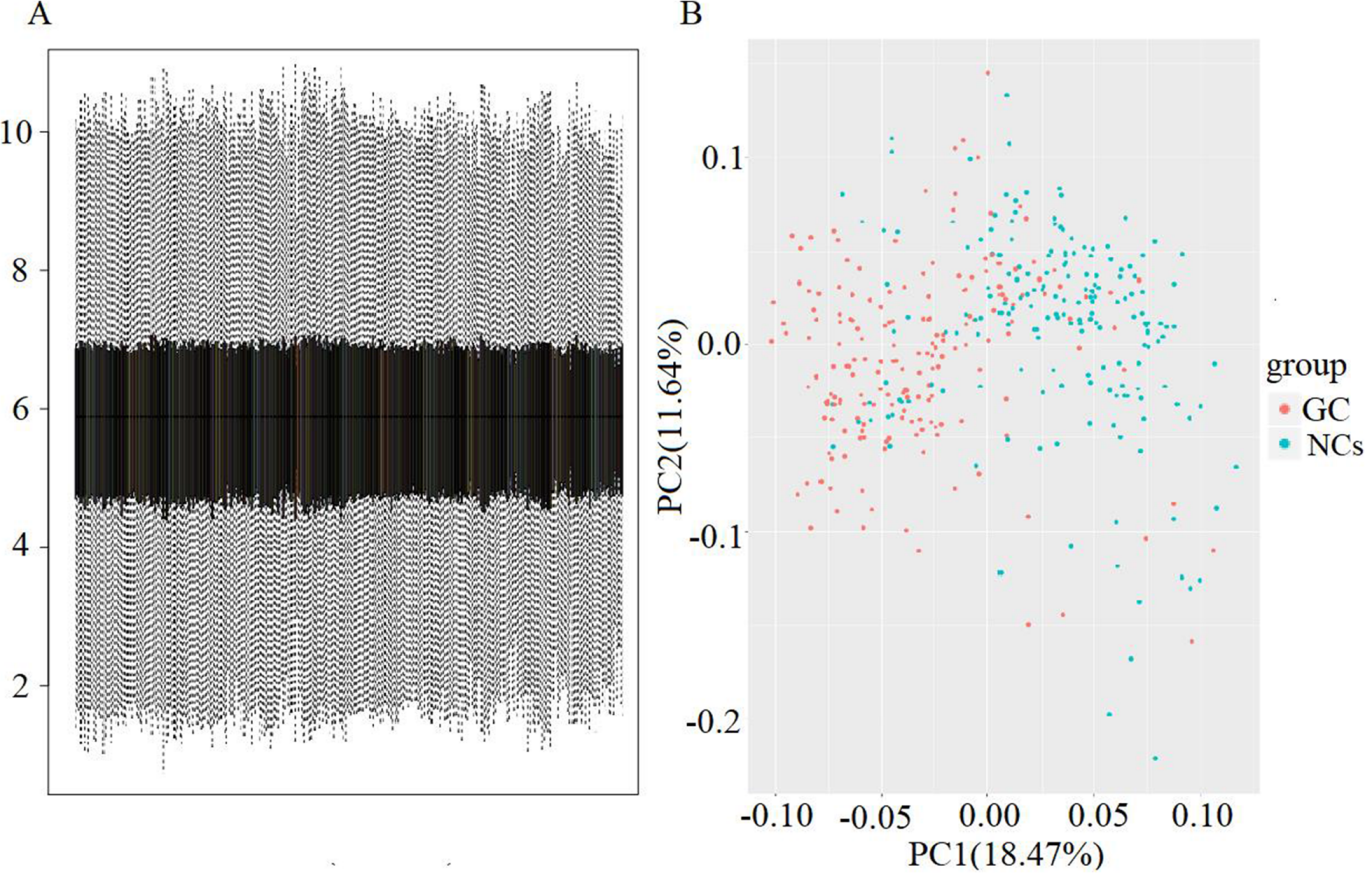
Data normalization and the distribution of differentially expressed genes (DEGs). (A) Box plots illustrating data normalization: the data distributions were neat after background adjustment and normalization. (B) Principal component analysis (PCA): each point in the PCA diagram represents a sample, and the distance between samples reflects the difference. After batch correction, individuals with similar genetic background were clustered together, and obvious stratification was observed between GC and adjacent normal gastric tissue samples. Gastric cancer, GC. normal controls, NCs

**Fig. 3:**
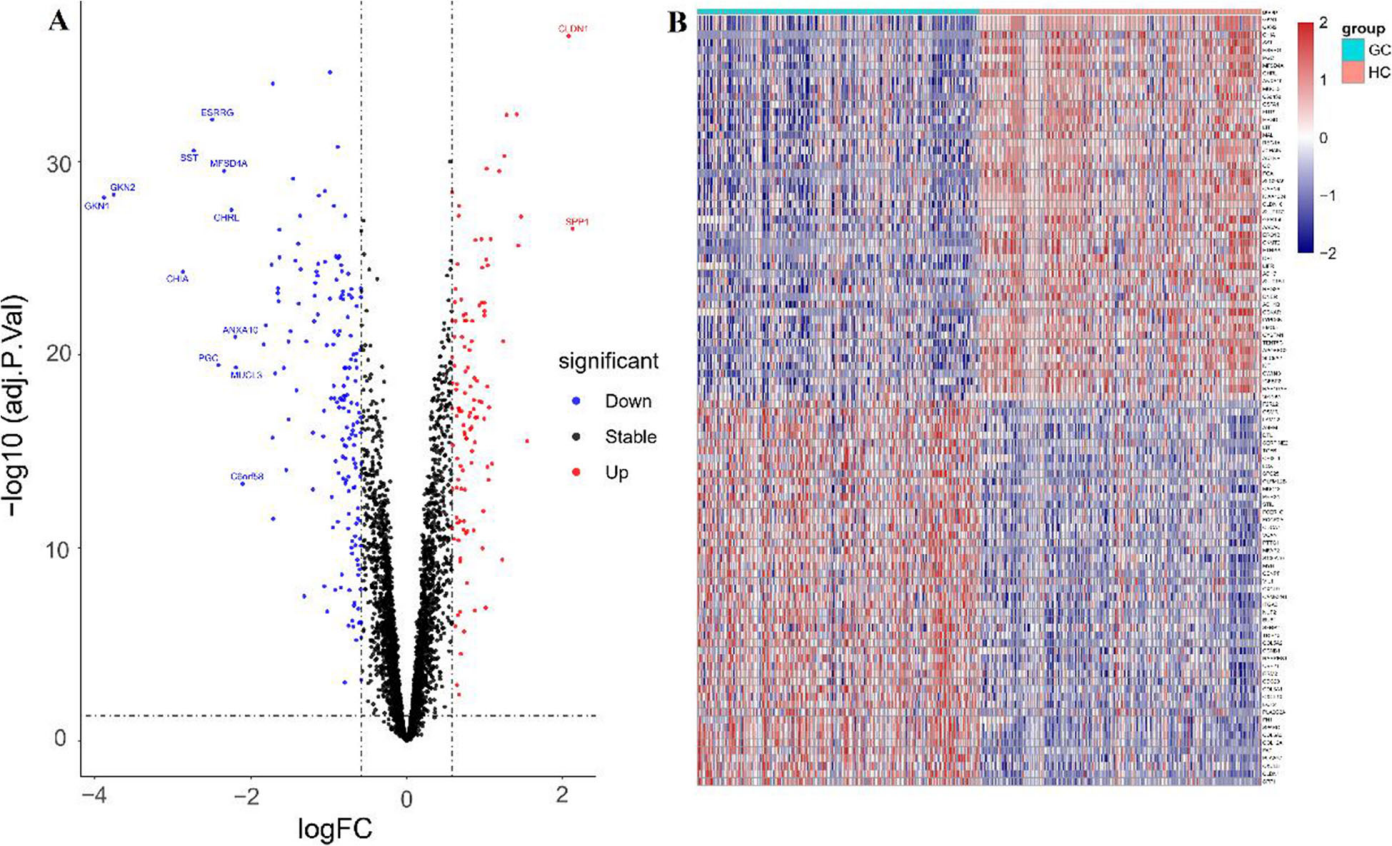
Volcano plot and heatmap of DEGs. (A) Volcanic map of DEGs: each colored dot represents a DEG based on the criteria of *P* < 0.05 and |log FC| > 0.58; red: up-regulation, blue: downregulation, black: normally expressed mRNAs. (B) Heatmap of top 50 significant up-regulated and down-regulated DEGs expressed in mRNAs microarrays. The horizontal axis shows clusters of DEGs, and the right vertical axis represents each sample. Gene expression levels were indicated by colors: red: high expression level and blue: low expression level

A total of 366 samples were included in the present study: 183GC and 183 adjacent normal tissues used as normal controls (NCs). Via R software, a total of 3224 DEGs (gastric cancer tissues vs. NCs), including 117 up-regulated and 178 down-regulated genes were selected. The statistical metrics for key DEGs was shown in Supplemental Table 1. The data distributions were neat after background adjustment and normalization with the RMA method, and values with an unchanged position in the boxplot were used for subsequent analysis.

(Figure 2A). Principal component analysis (PCA) was conducted to obtain better insights into the data. The DEGs of GC and normal tissues were relatively well separated in 2D score plot PCA. (Fig. 2B) The volcano plots of DEGs were shown in Fig.3A. DEGs expression heatmaps of the top 50 significant up-regulated genes and top 50 significant down-regulated genes were depicted in Fig. 3B, and hierarchical clustering analysis revealed that DEGs can be easily distinguished from GC tissues and normal gastric tissues.

### PPI and modular analysis

**Fig. 4:**
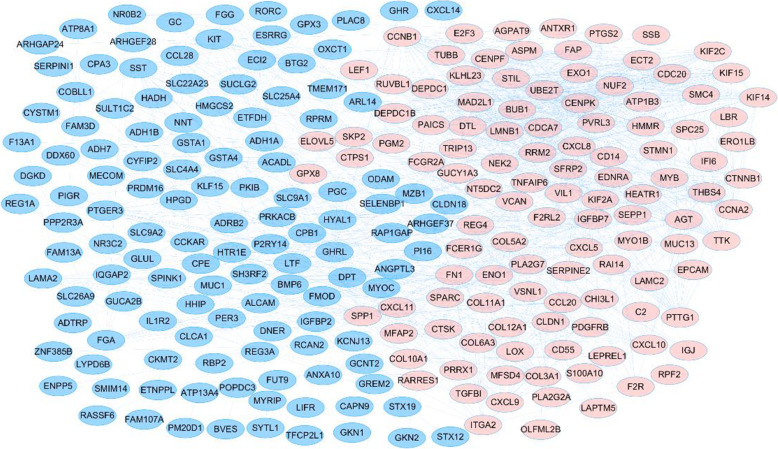
PPI network of the DEGs in GC. The PPI network of DEGs was constructed using Cytoscape. The nodes meant proteins; the edges meant the interaction of proteins. Upregulated genes are marked in light red; downregulated genes are marked in blue. PPI: protein–protein interaction; DEG: differentially expressed gene; GC: gastric cancer

**Fig. 5:**
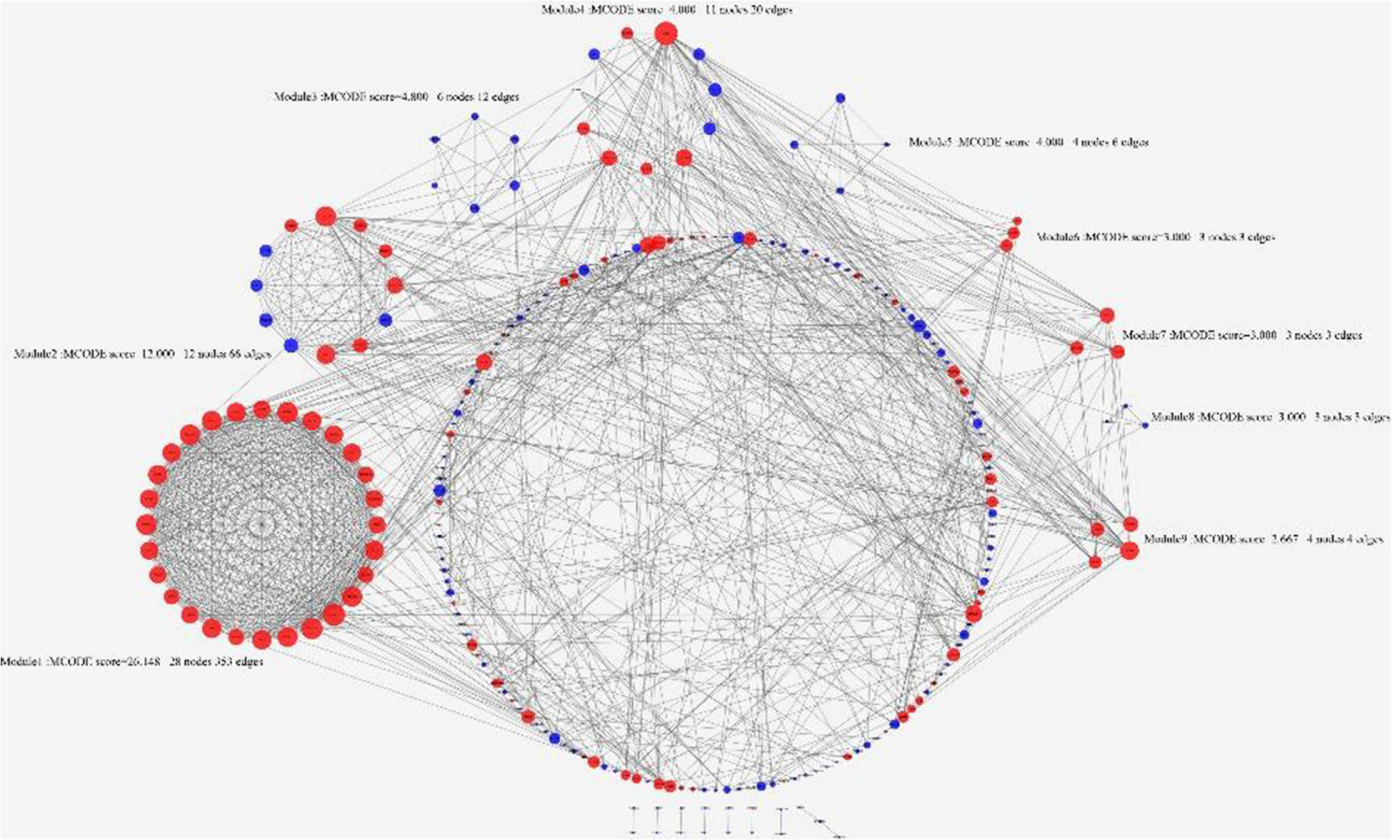
The 9 modules identified in the PPI network. Module analysis via Cytoscape software (degree cutoff = 2, node score cutoff = 0.2, k-core = 2, and max. Depth = 100). Node size represents the degree score; lines represent interactions

Based on the STRING online database, a total of 295 DEGs were imported into the DEG PPI network complex which included 291 nodes and 1016 edges. All the parameters were set as defaults [10]. The average node degree of PPI network was 6.98 and the local clustering coefficient was 0.446. To further investigate the PPI, the PPI network was visualized by cytoscape. (Fig. 4) Nine modules were exhibited after analyzing the entire PPI network by MCODE plug-in (Fig. 5& Supplemental Table 2).

### Identification of the selected genes

**Fig. 6:**
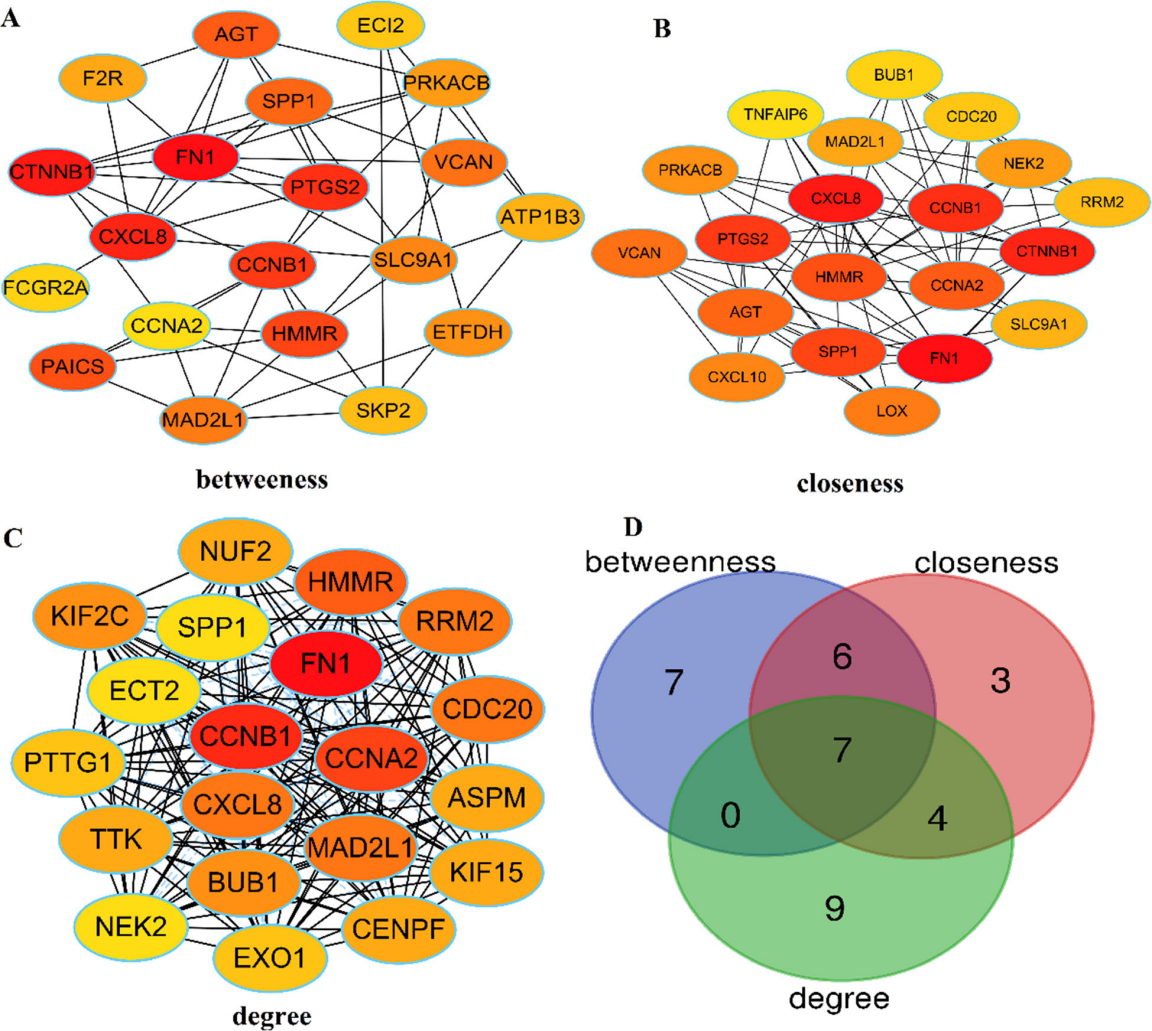
Seven hub genes selected from PPI network. (A) Hub genes screened by betweenness centrality according to cytoHubba plug-in. (B) Hub genes screened by closeness according to cytoHubba plug-in. (C) Hub genes screened by degree according to cytoHubba plug-in. (D) Venn diagram of DEGs. Hub genes were HMMR, SPP1, FN1, CCNB1, CXCL8, MAD2L1, and CCNA2; PPI, protein–protein interaction

The vital genes were determined from the PPI network by cytohubba plug-in. All the gene code and edge were calculated (Fig.6A.B.C& Supplemental Table 3). Three groups of DEGs calculated from degree, betweenness centrality and closeness were subjected to VEEN analysis (Fig.6D& Supplemental Table 3 & Supplemental Table 4). The overlapping genes were sequentially listed as follows: HMMR (hyaluronan mediated motility receptor), SPP1 (secreted phosphoprotein 1), FN1(fibronectin 1), CCNB1 (cyclin B1), CXCL8 (C-X-C motif chemokine ligand 8), MAD2L1 (mitotic arrest deficient 2 like 1), CCNA2 (cyclin A2). (Table 2) Besides, the selected genes also showed significant enrichment in modules by MCODE analysis (Fig. 5). Some of these genes exhibited potential prognostic values for patients with GC.

**Table 2:** Details of seven selected genes

N	Gene symbol	Full name	Function
1	HMMR	hyaluronan mediated motility receptor	the driver of tumor progression, plays a important role in the modulation of motor activities and the maintenance of genome stability.
2	SPP1	secreted phosphoprotein 1	The protein encoded by this gene is involved in the attachment of osteoclasts to the mineralized bone matrix
3	FN1	fibronectin 1	Fibronectin 1 (FN1) is involved in cell adhesion and migration processes including embryogenesis, wound healing, blood coagulation, host defense, metastasis, and implicated in various biochemical processes.
4	CCNB1	cyclin B1	an important regulator in cell cycle machinery, is a monitoring protein in mitosis and expressed primarily in G2/M phase which is critical for controlling the cell cycle at the G2/M (mitosis) transition
5	CXCL8	C-X-C motif chemokine ligand 8	a chemokine that acts as an important multifunctional cytokine to modulate tumour proliferation, invasion and migration in an autocrine or paracrine manner.
6	MAD2L1	mitotic arrest deficient 2 like 1	a component of the mitotic spindle assembly checkpoint that prevents the onset of anaphase until all chromosomes are properly aligned at the metaphase plate
7	CCNA2	cyclin A2	The protein encoded by this gene belongs to the highly conserved cyclin family, whose members function as regulators of the cell cycle.

### Survival analysis of selected genes by the Kaplan Meier plotter and GEPIA

**Fig. 7:**
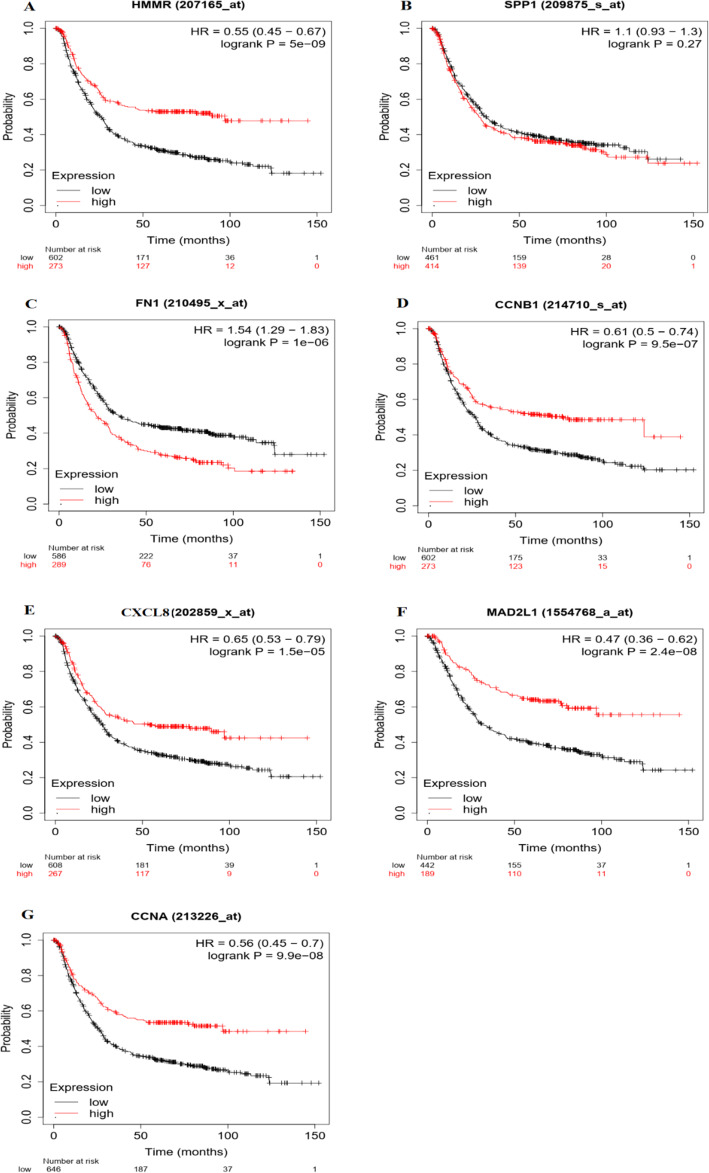
Genes associated with patient’s survival outcomes by applying the K-M method. Prognostic curves of most selected genes showed a significantly worse survival rate (*P* < 0.05). The red lines represent patients with high gene expression, and black lines with a low gene expression. HR: hazard ratio.

**Fig. 8:**
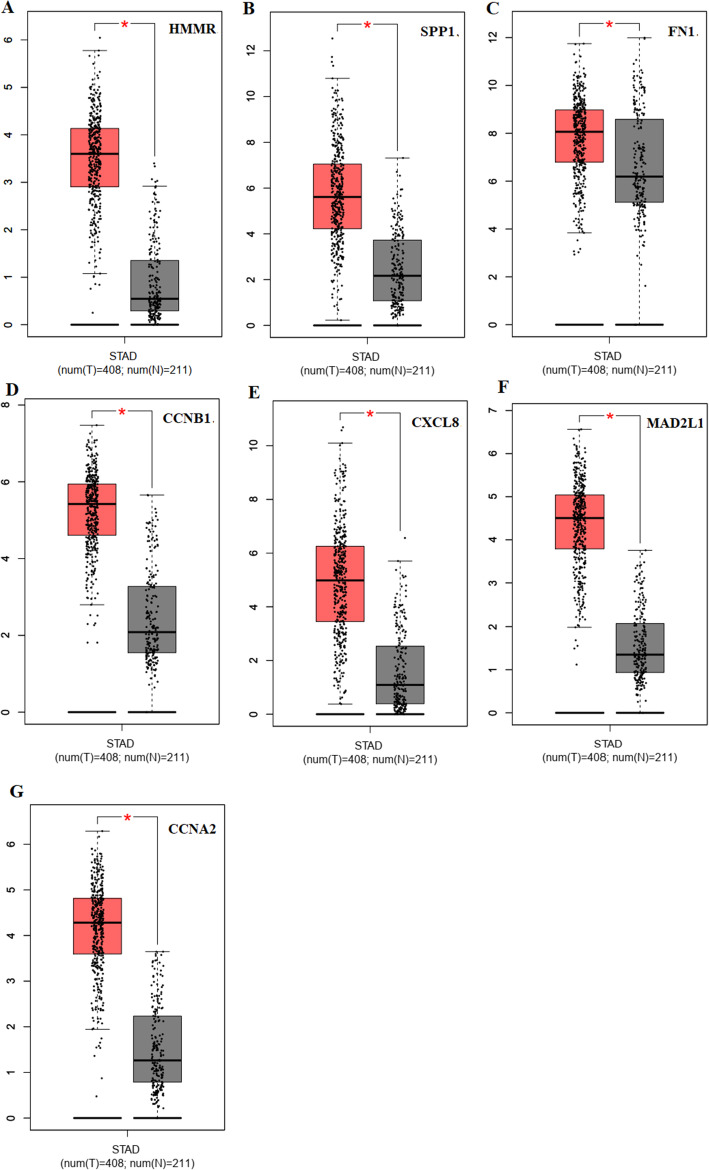
Differentially expressed genes related with poor prognosis were analyzed using GEPIA website. These genes had significantly upregulated expression in gastric cancers compared to normal specimen (*P < 0.05). The red and gray boxes represent cancer and normal tissues, respectively. STAD: Stomach adenocarcinoma

To further analyze the prognostic value of the selected genes, the overall survivals (OS) with selected genes were analyzed for 875 patients with GC by using the Kaplan-Meier plotter. It was found that most of the genes had a significantly worse survival (*P* < 0.05, Fig.7). High expression of HMMR (*P = 5.0e-9*), FN1 (*P = 1.0e-6*), CCNB1(*P = 9.5e-7*), CXCL8(*P* = 1.5e-5), MAD2L1(*P* = 2.4e-8), CCNA2(*P = 9.9e-8)* were correlated with significantly worse OS in GC patients, while SPP1 expression was not relevant to survival (*P* = 0.2713). Then, we used GEPIA to dig up the expression levels of selected genes in GC patients and healthy controls. Results reflected that, contrasted to normal samples, all the selected genes reflected high expressed in GC samples (*P* < 0.05, Fig. 8).

### Transcriptional factor regulatory network analysis of selected genes

**Fig. 9:**
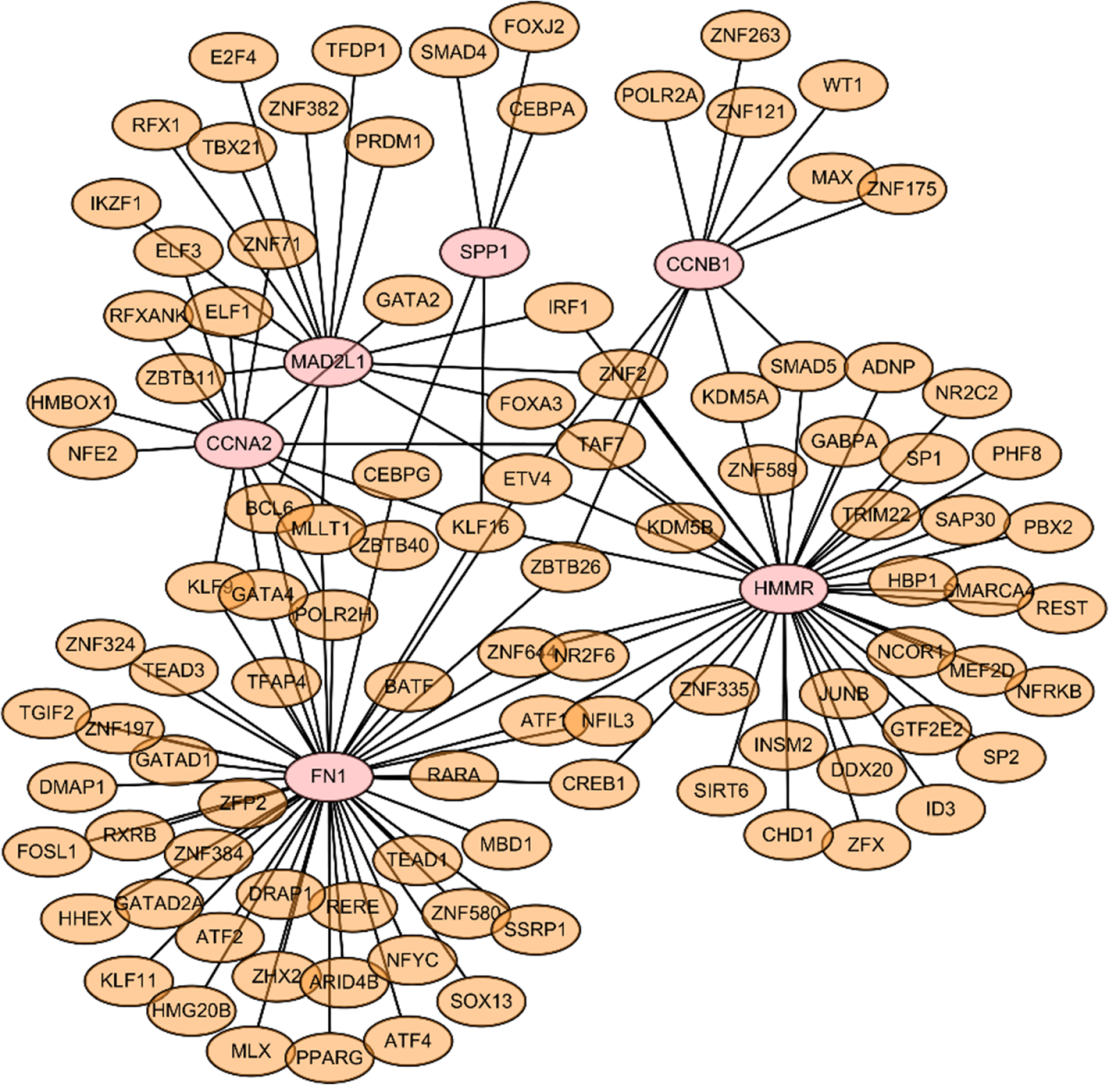
The hub gene-transcription factor (TF) regulatory network. Pink circle stands for the hub gene and orange node stands for the transcription factor

For the genes we identified, a gene-TF regulatory network was constructed including 129 interaction pairs among the selected genes and 102 TFs (Fig. 9 & Supplemental Table 5). While HMMR was found to be regulated by 39 TFs, SPP1 by 5 TFs, FN1 by 45 TFs, CCNB1 by 11 TFs, and CCNA2 by 14 TFs. In addition, various TFs were found to regulate more than one hub gene, and twenty TFs were identified with a connectivity degree ≥ 2 in the gene-TF regulatory network, which means that these TFs have close interactions with these hub DEGs. For example, zinc finger protein 2 (ZNF2) was predicted to regulate HMMR, and MAD2L1; ETS variant transcription factor 4 (ETV4) was found to regulate HMMR, FN1, MAD2L1, and CCNB1; Kruppel like factor 16 (KLF16) was found to regulate HMMR, SPP1, FN1, and CCNA2.

### Analysis of 7 selected genes via gene ontology and pathway enrichment

To understand the possible pathway of these 7 selected DEGs, KEGG pathway enrichment was re-analyzed via DAVID (P < 0.05). GO analysis revealed 7 selected genes that are involved in a number of biological processes (BP), including positive regulation of fibroblast proliferation, cell division, and negative regulation of ubiquitin-protein ligase activity involved in mitotic cell cycle. In terms of cellular components, 7 selected genes were mostly enriched in spindle pole, extracellular space, and extracellular region. The 7 selected genes were mainly associated with protein binding in terms of molecular functions. With regards to the KEGG pathway analysis of the 7 selected genes, ten pathways were enriched: ‘ECM-receptor interaction’, ‘Progesterone-mediated oocyte maturation’, ‘Cell cycle’, ‘Amoebiasis’, ‘Toll-like receptor signaling pathway’, and ‘Oocyte meiosis’. Detailed results are displayed in Table 3.These results suggested that Toll-like receptor signaling pathway and Cell cycle played extremely important roles in progesterone resistance and should be further studied.

**Table 3:** Analysis of 7 selected genes by GO and KEGG pathway enrichment. FDR, false discovery rate. GO, gene ontology; BP, biological process, CC, cellular component; MF, molecular function; KEGG, Kyoto Encyclopedia of Genes and Genomes; DEMs, differentially expressed miRNAs

Category	Term	Count	%	PValue	Genes	FDR
GOTERM_BP_DIRECT	GO:0048146 ~ positive regulation of fibroblast proliferation	3	0.263	1.51E-04	CCNB1, CCNA2, FN1	0.174735
GOTERM_BP_DIRECT	GO:0051301 ~ cell division	3	0.263	0.006147477	CCNB1, MAD2L1, CCNA2	6.892135
GOTERM_BP_DIRECT	GO:0051436 ~ negative regulation of ubiquitin-protein ligase activity involved in mitotic cell cycle	2	0.175	0.025106263	CCNB1, MAD2L1	25.50655
GOTERM_BP_DIRECT	GO:0051437 ~ positive regulation of ubiquitin-protein ligase activity involved in regulation of mitotic cell cycle transition	2	0.175	0.026854324	CCNB1, MAD2L1	27.03882
GOTERM_BP_DIRECT	GO:0022617 ~ extracellular matrix disassembly	2	0.175	0.026854324	SPP1, FN1	27.03882
GOTERM_BP_DIRECT	GO:0031145 ~ anaphase-promoting complex-dependent catabolic process	2	0.175	0.027901906	CCNB1, MAD2L1	27.94322
GOTERM_BP_DIRECT	GO:0071456 ~ cellular response to hypoxia	2	0.175	0.033820471	CCNB1, CCNA2	32.86331
GOTERM_BP_DIRECT	GO:0000086 ~ G2/M transition of mitotic cell cycle	2	0.175	0.047971221	CCNB1, HMMR	43.40814
GOTERM_BP_DIRECT	GO:0042787 ~ protein ubiquitination involved in ubiquitin-dependent protein catabolic process	2	0.175	0.053446406	CCNB1, MAD2L1	47.06463
GOTERM_BP_DIRECT	GO:0030198 ~ extracellular matrix organization	2	0.175	0.068031098	SPP1, FN1	55.77698
GOTERM_BP_DIRECT	GO:0001525 ~ angiogenesis	2	0.175	0.077092848	CXCL8, FN1	60.50819
GOTERM_CC_DIRECT	GO:0000922 ~ spindle pole	2	0.175	0.035359174	CCNB1, MAD2L1	26.96429
GOTERM_CC_DIRECT	GO:0005615 ~ extracellular space	3	0.263	0.067058677	CXCL8, SPP1, FN1	45.44016
GOTERM_CC_DIRECT	GO:0005576 ~ extracellular region	3	0.263	0.092078284	CXCL8, SPP1, FN1	56.9647
GOTERM_MF_DIRECT	GO:0005515 ~ protein binding	7	0.613	0.019847501	CCNB1, MAD2L1, CXCL8, CCNA2, SPP1, FN1, HMMR	13.51086
KEGG_PATHWAY	hsa04512:ECM-receptor interaction	3	0.263	0.002294922	SPP1, FN1, HMMR	2.054976
KEGG_PATHWAY	hsa04914:Progesterone-mediated oocyte maturation	3	0.263	0.002294922	CCNB1, MAD2L1, CCNA2	2.054976
KEGG_PATHWAY	hsa04110:Cell cycle	3	0.263	0.004611117	CCNB1, MAD2L1, CCNA2	4.090835
KEGG_PATHWAY	hsa05146:Amoebiasis	2	0.175	0.088997082	CXCL8, FN1	56.93087
KEGG_PATHWAY	hsa04620:Toll-like receptor signaling pathway	2	0.175	0.088997082	CXCL8, SPP1	56.93087
KEGG_PATHWAY	hsa04114:Oocyte meiosis	2	0.175	0.093026279	CCNB1, MAD2L1	58.62208

### The transcription levels of selected genes were verified within GC tissues

**Fig. 10:**
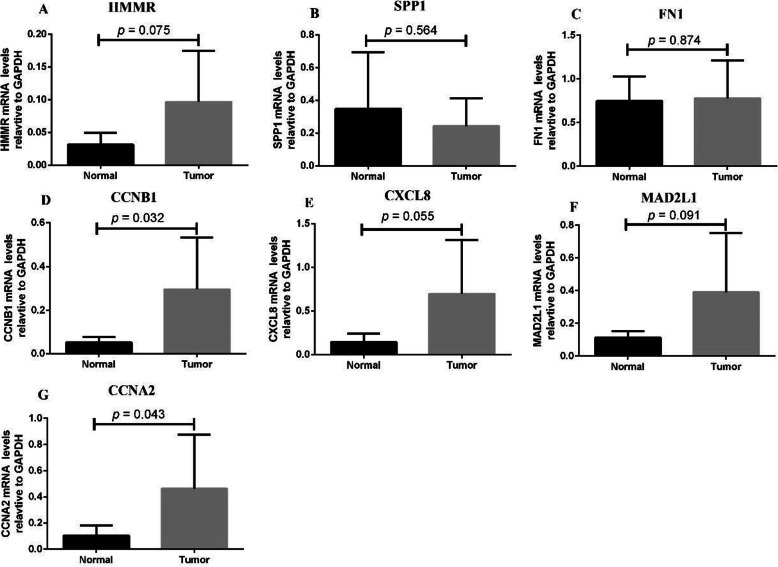
Validation of 7 selected gene expression in gastric cancer samples was performed by qRT-PCR analysis. (A) HMMR; (B) SPP1; (C) FN1; (D) CCNB1; (E) CXCL8; (F) MAD2L1; (G) CCNA2. Expression of these DEGs was normalized against GAPDH expression. The statistical significance of differences was calculated by the Student’s t-test

To further verify the results of bioinformatics analysis, we applied qRT-PCR to validate the mRNA levels of HMMR, SPP1, FN1, CCNB1, CXCL8, MAD2L1 and CCNA2 in 10 paired tumor and adjacent normal tissues with qRT-PCR. Among the genes we validated, HMMR, CCNB1, CXCL8, MAD2L1, and CCNA2 showed increasing expression levels in GC. As illustrated in Fig. 10, high expression of CCNB1 and CCNA2 significantly correlates with tumor relapse and predicts poorer prognosis in GC patients (*P* < 0.05). The expression of HMMR, CXCL8 and MAD2L1 shows an increasing trend in GC, whereas COL1A2 and SPP1 expression levels might not affect the prognosis of patients with GC. We identified 5 hub genes including HMMR, CCNB1, CXCL8, MAD2L1, and CCNA2 with poor prognosis in GC on the basis of integrated bioinformatical methods, which could be potential biomarkers and therapeutic targets for GC treatment.

## Discussion

GC is a gastroenterological malignancy with high rates of prevalence and mortality [1, 2, 17, 18]. Therefore, sensitive and specific biomarkers of GC are urgently needed to be detected. In the present study, bioinformatic methods are promising methods to analyze the critical genes and pathways, which might provide novel clues for diagnosis, therapy, and prognosis of GC. We integrated seven gene expression profile datasets from different groups and used R software and bioinformatics to deeply analyze these datasets. DEGs PPI network was successfully constructed via the STRING online database and cytoscape software. Seven vital regulated genes including HMMR, SPP1, FN1, CCNB1, CXCL8, MAD2L1, and CCNA2 were screened from the PPI network complex by cytohubba plug-in of cytoscape.

Through Kaplan Meier plotter analysis, we found that most of the selected genes were associated with a significantly worse survival, except SPP1. The expression of the genes was higher in GC samples than normal samples by GEPIA analysis. Importantly, using qRT-PCR, we could validate the higher mRNA expression of the selected genes based our bioinformatics analysis; most selected genes, except SPP1 and FN1, were upregulated in tumor tissue. They showed the same trend in expression as predicted by bioinformatics verifying the accuracy of our method. In the light of important roles in cells, the selected hub genes in GC (HMMR, CCNB1, CXCL8, MAD2L1, and CCNA2) may represent potential prognostic biomarkers and/or therapeutic targets for GC.

For a more in-depth understanding of these DEGs, we analyzed the selected genes for GO and KEGG enrichment analyses and found that ‘Cell cycle’ signaling pathways was significant enriched. HMMR, CCNB1, MAD2L1 and CCNA2 play important roles in cell cycle. HMMR, a cell surface hyaluronan receptor and mitotic spindle protein and the driver of tumor progression [19] [20] [21], plays an important role in the modulation of motor activities and the maintenance of genome stability [22, 23]. High expression of HMMR significantly correlates with tumor relapse [24, 25] and predicts poorer prognosis in GC patients. Furthermore, HMMR has been identified as a promising target for antibody therapy to block the extracellular function of HMMR on the surface of tumor cells [26], which might be a potential prognostic marker or therapeutic target against the disease. The protein encoded by CCNB1 gene is an important monitoring protein in mitosis, which is necessary for proper controlling the cell cycle at the G2/M transition phase [27]. Previous studies have reported that the CCNB1–Cdk1 complex is a key regulator of mitotic entry [28]. Recently, increasing evidence demonstrated that CCNB1 was over-expressed in considerable cancers with poor prognosis, including hepatocellular carcinoma [29, 30], breast cancer [31, 32], and pancreatic cancer [33, 34]. The expression of CCNB1 is often used to estimate prognosis after treatment with anticancer drugs [29, 35]. Studies had shown that CCNB1 were associated with gastric cancer [36, 37]. HnRNPR-CCNB1/CENPF axis may be a potential therapeutic target for GC treatment [38].

The function of MAD2L1 is to maintain the separation state of chromosomes during the dissociation of mitotic chromosomes and spindle, and to play a role in the checkpoint during mitosis [39, 40]. Abnormal regulation of MAD2L1 is associated with chromosomal instability and a large number of aneuploidy, which can lead to tumorigenesis [41]. Studies have found that MAD2L1 is overexpressed in lung adenocarcinoma tissues, and the overexpression of MAD2L1 may indicate poor prognosis and increased risk of tumor recurrence in patients, which can be used as a prognostic marker for lung adenocarcinoma [39]. Our bioinformatics analysis showed that MAD2L1 was highly expressed in tumor tissues compared with normal tissues. MAD2L1 is a pro-oncogene which is upregulated in GC [42, 43], and we need to further study its specific mechanism. The protein encoded by CCNA2 belongs to the highly conserved cyclin family, whose members function as regulators of the cell cycle at the G1/S and the G2/M transitions [44]. CCNA2 is overexpressed in several human cancers and closely related to tumor progression and shorter survival in lung, breast, and colorectal cancer [45–49]. Poor prognosis in GC patients related with high expressions of cyclins [50].CCNA2 is a novel predictive biomarker of sensitivity to PLK1 inhibitors for the treatment of advanced gastric cancer [51], whose overexpression was an indicator of poor prognosis. Limited by few studies about evaluating the expression and prognostic role of CCNA2 in GC patients, more efforts are necessary to confirm expression pattern and prognostic role of CCNA2 in GC patients.

CXCL8 is a member of the CXC chemokine family that acts as an important multifunctional cytokine to modulate tumor proliferation, invasion and migration in an autocrine or paracrine manner. Neovascularization, which provides a basis for fostering tumor growth and metastasis, is now recognized as a critical function of CXCL8 in the tumor microenvironment [52]. CXCL8 signaling axis also plays an indispensable role in colorectal carcinoma [53, 54], renal cell carcinoma, pancreatic cancer, thyroid tumors, gastric cancer [55, 56], and lymphomas [57]. Aberrant activation of CXCL8 in cancer-associated fibroblasts is correlated with poorer survival in gastric cancer patients [58]. Microarray analysis revealed that protein tyrosine phosphatase receptor delta-inactivation-induced CXCL8 promotes angiogenesis and metastasis in gastric cancer [59]. Interruption of the related signaling pathways may thus provide promising therapeutic avenues for tumors. Studies have found that CXCL8 is predominantly secreted by macrophages and contributes to the immunosuppressive microenvironment by inducing PD-L1+ macrophages in GC [60]. CXCL8 could be an early detection marker for perineural invasion-related GC, with a potential to be utilized as individual therapy targets [61]. CXCL8 inhibitors may drive antitumor response, providing potential therapeutic effects for patients with gastric cancer.

To further screen the TFs in hub genes, we constructed a gene-TF regulatory network and found IRF1, ETV4, KLFs, and SMAD5 that were meaningful in GC. It was reported that MTMR2 mediated epithelial-mesenchymal transition through the IFNγ/STAT1/IRF1 pathway to promote GC invasion and metastasis [62]. KIF2A expression is a potential target for GC therapy, which can be upregulated by transcription factor ETV4 [63]. Krüppel-like factors (KLFs) have been extensively investigated in multi-cancers, which plays a significant role in GC progression and could be a new therapeutic target for GC patients. Interestingly, SMAD5 was frequently altered in human GC [64]. The intricate interaction between TFs and other hub genes made great contribution to the development of cancer.

Studied have proved that Toll-like receptor (TLR) signaling pathways play important roles in development of GC. TLR signaling pathways are involved in innate and adaptive immunity responses [65] and activation of both inflammatory and carcinogenic processes [66]. Thus, the pattern of the host’s immune response beyond genetic and environmental factors is also essential for understanding the pathology of GC [67]. TLRs, a class of transmembrane receptors [68], play an important role in defense against Helicobacter pylori (*H. pylori*) widely known as a class I carcinogen in GC [68]. Therefore, the abnormal expression of TLRs is closely related to tumorigenesis and cancer progression and a better understanding of TLRs will provide new diagnostic or predictive markers for the diagnosis of GC.

We failed to validate SPP1 as a DEG in our fresh GC samples, which may be as a result of the small sample size and inter-sample variation. The protein encoded by SPP1 plays an important role in tumorigenesis, invasion and metastasis [10, 69]. Overexpressed SPP1 expression had been confirmed in various types of cancers [70–73]. A study based on gastric cancer cell lines indicated that the elevated expression of SPP1 is a critical determinant of poor prognosis [74]. In addition, in a recent study, SPP1 rs4754 polymorphism was observed to be associated with the risk of gastric cancer and has an important effect in gastric carcinogenesis [75]. However, it has been reported that SPP1 might not affect the prognosis of patients with GC [73], which needs more study in the future.

All above, we found that high expression of 5 validated hub genes should promote the progress of GC patients, suggesting that their antagonism may improve the prognosis of GC. Although some of these genes were found before, our study could validate and explain the expression status of these genes and their impact on prognosis in GC again. These findings provide a set of useful driving genes and key pathways of cancers, which are worth future investigating for novel therapeutic targets, a prognostic evaluation index, and the detailed pathogenesis of them in GCs.

However, there were several limitations of the present study. Firstly, validation with qRT-PCR study need more tumor and adjacent normal tissues samples. Second, more experiments, such as immunohistochemistry and Western blot, should be conducted to confirm the protein levels in GC.

## Conclusion

Taken above, our bioinformatics analysis identified 295 DEGs of GC. Among them, HMMR, CCNB1, CXCL8, MAD2L1 and CCNA2 were verified and considered as Hub genes were associated with disease prognosis, which could be predictive and therapeutic targets.

## Supplementary Information

**Additional file 1 Table S1.** All 295 commonly DEGs were detected from seven profile datasets, including 178 down-regulated genes and 117 up-regulated genes in the GC tissues compared to normal gastric tissues. **Table S2.** Significant models were obtained from the PPI network based on the MCODE analysis in Cytoscape. **Table S3.** The determined selected genes by using the cytoHubba plugin such as degree, betweenness centrality, and closeness. **Table S4.** The determined selected genes of Venn diagram. **Table S5.** The gene-TF regulatory network was constructed including 129 interaction pairs among 7 genes and 102 TFs.

## Data Availability

All data generated or analyzed during this study are included in this published article.

## Abbreviations

GC: Gastric cancer
DEGs: Differentially expressed genes
GEPIA: Gene Expression Profiling Interactive analysis
KEGG: Kyoto Encyclopedia of Gene and Genome
PPI: protein–protein interaction
TF: Transcriptional Factor
qRT-PCR: quantitative real-time PCR
RMA: Robust Multi-array Average
BH: Benjamini & Hochberg
MCODE: Molecular complex detection
DAVID: Database for annotation, visualization and integrated discovery
cDNA: complementary DNA
NCs: Normal Controls
PCA: Principal component analysis
HMMR: Hyaluronan Mediated Motility Receptor
SPP1: Secreted phosphoprotein 1
FN1: Fibronectin 1
CCNB1: Cyclin B1
CXCL8: C-X-C motif chemokine ligand 8
MAD2L1: Mitotic arrest deficient 2 like 1
CCNA2: cyclin A2
OS: Overall Survivals
ZNF2: Zinc finger protein 2
KLF16: Kruppel like factor 16
BP: Biological Processes
KLFs: Krüppel-like factors
TLR: Toll-like receptor
*H. pylori*: Helicobacter pylori
