# Supplementary material for: Identification of novel hub genes associated with gastric cancer using integrated bioinformatics analysis
Source: BMC Cancer. 2021 Jun 14;21:697. doi: 10.1186/s12885-021-08358-7 (PMC8201699; doi:10.1186/s12885-021-08358-7)
Supplement: Supplementary file 1 — Additional file 1 Table S1. All 295 commonly DEGs were detected from seven profile datasets, including 178 down-regulated genes and 117 up-regulated genes in the GC tissues compared to normal gastric tissues. Table S2. Significant models were obtained from the PPI network based on the MCODE analysis in Cytoscape. Table S3. The determined selected genes by using the cytoHubba plugin such as degree, betweenness centrality, and closeness. Table S4. The determined selected genes of Venn diagram. Table S5. The gene-TF regulatory network was constructed including 129 interaction pairs among 7 genes and 102 TFs. [file 12885_2021_8358_MOESM1_ESM.docx]

**Supplemental Table1:** All 295 commonly DEGs were detected from seven profile datasets, including 178 down-regulated genes and 117 up-regulated genes in the GC tissues compared to normal gastric tissues.

| Gene_Symbol | logFC | AveExpr | t | P.Value | adj.P.Val | group |
| --- | --- | --- | --- | --- | --- | --- |
| CLDN1 | 2.079070349 | 5.822104591 | 15.20927389 | 6.81E-41 | 3.08E-37 | up |
| COL12A1 | 1.41168492 | 6.782880698 | 14.05650265 | 3.08E-36 | 3.47E-33 | up |
| COL6A3 | 1.282522225 | 8.224752865 | 14.02758864 | 4.01E-36 | 3.63E-33 | up |
| SPARC | 1.256562042 | 9.392415768 | 13.42205159 | 1.01E-33 | 5.08E-31 | up |
| COL5A2 | 1.027501146 | 7.090841497 | 13.23332945 | 5.57E-33 | 2.29E-30 | up |
| ECT2 | 1.187573744 | 5.748673221 | 13.18851965 | 8.35E-33 | 3.05E-30 | up |
| HEATR1 | 0.585950869 | 6.386223412 | 12.87687317 | 1.37E-31 | 3.86E-29 | up |
| PGM2 | 0.673864366 | 6.484664864 | 12.66578977 | 8.97E-31 | 1.93E-28 | up |
| RUVBL1 | 0.668215326 | 6.155085623 | 12.51328398 | 3.46E-30 | 6.34E-28 | up |
| PLA2G7 | 1.468773763 | 5.730397685 | 12.4948808 | 4.08E-30 | 7.08E-28 | up |
| SPP1 | 2.130579253 | 6.235406933 | 12.32178901 | 1.87E-29 | 3.02E-27 | up |
| MFAP2 | 0.960242678 | 5.945508016 | 12.16797062 | 7.21E-29 | 1.05E-26 | up |
| COL3A1 | 1.079522626 | 9.454607473 | 12.16354015 | 7.49E-29 | 1.06E-26 | up |
| STIL | 0.883187843 | 4.845813316 | 12.15054515 | 8.39E-29 | 1.15E-26 | up |
| FAP | 1.434687911 | 4.236416326 | 12.06693483 | 1.74E-28 | 2.25E-26 | up |
| TRIP13 | 1.020903833 | 5.333846783 | 11.85785647 | 1.07E-27 | 1.18E-25 | up |
| RAI14 | 0.652553471 | 6.082746706 | 11.78419576 | 2.02E-27 | 1.98E-25 | up |
| UBE2T | 1.041371364 | 5.683976241 | 11.75888309 | 2.51E-27 | 2.31E-25 | up |
| CENPF | 0.969981978 | 5.151011698 | 11.72638052 | 3.32E-27 | 3.00E-25 | up |
| E2F3 | 0.628639928 | 5.340969414 | 11.39664901 | 5.56E-26 | 3.86E-24 | up |
| MYO1B | 0.700413469 | 6.124601481 | 11.22391673 | 2.40E-25 | 1.39E-23 | up |
| CTPS1 | 0.618435309 | 5.897483014 | 11.18519767 | 3.32E-25 | 1.85E-23 | up |
| S100A10 | 0.961662974 | 11.11903724 | 11.17404602 | 3.65E-25 | 2.01E-23 | up |
| NUF2 | 0.998163722 | 4.410780274 | 11.17145883 | 3.73E-25 | 2.03E-23 | up |
| VCAN | 0.933460244 | 7.044858657 | 11.13223831 | 5.18E-25 | 2.76E-23 | up |
| BUB1 | 0.998373094 | 4.687293935 | 11.04722539 | 1.06E-24 | 5.43E-23 | up |
| F2R | 0.756760839 | 7.218923431 | 11.00355533 | 1.52E-24 | 7.65E-23 | up |
| STMN1 | 0.729421738 | 6.861155196 | 10.97999878 | 1.85E-24 | 9.10E-23 | up |
| ITGA2 | 0.997780752 | 6.16433791 | 10.97811107 | 1.88E-24 | 9.15E-23 | up |
| PDGFRB | 0.764474822 | 6.942437334 | 10.90342068 | 3.51E-24 | 1.67E-22 | up |
| ENO1 | 0.746713818 | 10.14939798 | 10.89771253 | 3.68E-24 | 1.73E-22 | up |
| TGFBI | 0.841332616 | 8.226745095 | 10.89403351 | 3.79E-24 | 1.77E-22 | up |
| KIF2A | 0.584186893 | 6.18984124 | 10.89102078 | 3.89E-24 | 1.79E-22 | up |
| FAM91A1 | 0.627893141 | 6.766310907 | 10.88686881 | 4.02E-24 | 1.82E-22 | up |
| SMC4 | 0.613524606 | 6.697272327 | 10.64514287 | 2.96E-23 | 1.20E-21 | up |
| LBR | 0.710494395 | 7.658987028 | 10.64254722 | 3.03E-23 | 1.20E-21 | up |
| FN1 | 1.23674502 | 8.162237539 | 10.57487779 | 5.27E-23 | 2.02E-21 | up |
| DTL | 0.836441461 | 5.109685737 | 10.57403548 | 5.31E-23 | 2.02E-21 | up |
| ATP1B3 | 0.743308081 | 7.126053489 | 10.48688986 | 1.08E-22 | 3.82E-21 | up |
| DEPDC1B | 0.808856028 | 4.917365758 | 10.43248112 | 1.68E-22 | 5.77E-21 | up |
| PAICS | 0.58443027 | 6.445945307 | 10.31915759 | 4.22E-22 | 1.39E-20 | up |
| NT5DC2 | 0.61517266 | 6.015397966 | 10.19058364 | 1.19E-21 | 3.73E-20 | up |
| KIF2C | 0.78547097 | 5.024400798 | 10.15676695 | 1.56E-21 | 4.70E-20 | up |
| EXO1 | 0.755240932 | 4.852001035 | 10.09419892 | 2.57E-21 | 7.35E-20 | up |
| OLFML2B | 0.870163602 | 5.483622457 | 9.990118559 | 5.89E-21 | 1.60E-19 | up |
| FCGR2A | 0.899955976 | 7.201036563 | 9.939337055 | 8.81E-21 | 2.27E-19 | up |
| CTNNB1 | 0.587963787 | 8.233128086 | 9.901668115 | 1.19E-20 | 2.95E-19 | up |
| SSB | 0.647142844 | 8.304094753 | 9.884368464 | 1.36E-20 | 3.34E-19 | up |
| OSMR | 0.822029874 | 6.165609583 | 9.84484319 | 1.86E-20 | 4.46E-19 | up |
| CCNB1 | 1.032618483 | 6.362975004 | 9.844402853 | 1.86E-20 | 4.46E-19 | up |
| CHN1 | 0.634874243 | 5.802615901 | 9.794622712 | 2.76E-20 | 6.49E-19 | up |
| EDNRA | 0.749100877 | 5.840023506 | 9.737769897 | 4.30E-20 | 9.77E-19 | up |
| SPC25 | 0.866017259 | 3.783827305 | 9.622490822 | 1.06E-19 | 2.23E-18 | up |
| PTTG1 | 0.934500054 | 7.536658931 | 9.599858818 | 1.26E-19 | 2.58E-18 | up |
| MYB | 0.961900594 | 5.295345731 | 9.584088188 | 1.43E-19 | 2.89E-18 | up |
| CDC20 | 1.058805839 | 5.935602963 | 9.496192284 | 2.81E-19 | 5.38E-18 | up |
| SKP2 | 0.666033649 | 6.597206824 | 9.481178002 | 3.16E-19 | 5.94E-18 | up |
| TUBB | 0.580629744 | 8.751310561 | 9.477413022 | 3.25E-19 | 6.09E-18 | up |
| IGFBP7 | 0.672618901 | 9.400993183 | 9.468002131 | 3.49E-19 | 6.50E-18 | up |
| LOX | 0.862312132 | 5.125324819 | 9.466011408 | 3.55E-19 | 6.57E-18 | up |
| KIF14 | 0.674392981 | 4.062313229 | 9.430553884 | 4.66E-19 | 8.42E-18 | up |
| LEF1 | 0.70508942 | 5.654995744 | 9.429938452 | 4.68E-19 | 8.43E-18 | up |
| F2RL2 | 0.817066044 | 4.394763415 | 9.405879366 | 5.63E-19 | 9.98E-18 | up |
| KIF15 | 0.643823447 | 3.865681453 | 9.351320869 | 8.55E-19 | 1.50E-17 | up |
| CCNA2 | 0.802525506 | 5.849554063 | 9.339302564 | 9.38E-19 | 1.62E-17 | up |
| CDCA7 | 0.900934121 | 6.585022782 | 9.32618769 | 1.04E-18 | 1.76E-17 | up |
| FCER1G | 0.88497092 | 6.830877336 | 9.316597448 | 1.11E-18 | 1.85E-17 | up |
| CAMK2N1 | 0.994163201 | 7.921148788 | 9.296676304 | 1.30E-18 | 2.12E-17 | up |
| ANTXR1 | 0.727387764 | 7.150322424 | 9.196038121 | 2.79E-18 | 4.37E-17 | up |
| ASPM | 0.835044994 | 4.393241794 | 9.160562064 | 3.64E-18 | 5.60E-17 | up |
| CTSK | 0.813967008 | 7.074133528 | 9.125560106 | 4.74E-18 | 7.12E-17 | up |
| NEK2 | 0.741768032 | 5.633439159 | 9.068709651 | 7.27E-18 | 1.06E-16 | up |
| TTK | 0.745533237 | 4.465834329 | 9.039291094 | 9.07E-18 | 1.30E-16 | up |
| DEPDC1 | 0.767577941 | 3.986516369 | 8.973227962 | 1.48E-17 | 2.06E-16 | up |
| GPX8 | 0.766530034 | 4.983130258 | 8.964060974 | 1.59E-17 | 2.18E-16 | up |
| CXCL8 | 1.547177115 | 7.22514081 | 8.923177448 | 2.16E-17 | 2.90E-16 | up |
| PRRX1 | 0.8754461 | 5.655389981 | 8.906082235 | 2.45E-17 | 3.28E-16 | up |
| ESM1 | 0.592858777 | 3.922890673 | 8.854351543 | 3.59E-17 | 4.76E-16 | up |
| SERPINE2 | 0.837497879 | 6.029540679 | 8.751974651 | 7.63E-17 | 9.63E-16 | up |
| LMNB1 | 0.649860357 | 6.015831826 | 8.625687788 | 1.92E-16 | 2.33E-15 | up |
| LAMC2 | 0.832178609 | 6.441745581 | 8.549328453 | 3.34E-16 | 3.95E-15 | up |
| CXCL10 | 1.095641709 | 6.079959924 | 8.53503213 | 3.70E-16 | 4.31E-15 | up |
| CENPK | 0.692109397 | 4.470150475 | 8.494404838 | 4.96E-16 | 5.73E-15 | up |
| RARRES1 | 1.036580455 | 5.701936399 | 8.479397107 | 5.53E-16 | 6.36E-15 | up |
| GASK1B | 0.72053291 | 6.778354025 | 8.416087659 | 8.71E-16 | 9.68E-15 | up |
| HMMR | 0.662987341 | 4.449785741 | 8.250691178 | 2.82E-15 | 2.92E-14 | up |
| RRM2 | 1.053497706 | 7.439855083 | 8.246305003 | 2.91E-15 | 3.00E-14 | up |
| KLHL23 | 0.643445799 | 5.191586164 | 8.107436119 | 7.72E-15 | 7.50E-14 | up |
| RPF2 | 0.701674356 | 6.949226936 | 8.088262073 | 8.82E-15 | 8.48E-14 | up |
| C2 | 0.58716082 | 6.143868248 | 8.07266623 | 9.83E-15 | 9.38E-14 | up |
| MAD2L1 | 0.747451624 | 5.829234126 | 8.067904143 | 1.02E-14 | 9.65E-14 | up |
| CXCL9 | 0.984634472 | 5.425116356 | 7.666154273 | 1.59E-13 | 1.28E-12 | up |
| LAPTM5 | 0.641016364 | 8.805263589 | 7.54161822 | 3.65E-13 | 2.77E-12 | up |
| AGT | 0.734330077 | 6.16570358 | 7.475292105 | 5.66E-13 | 4.19E-12 | up |
| TNFAIP6 | 0.682607946 | 4.789911512 | 7.473091134 | 5.75E-13 | 4.24E-12 | up |
| ELOVL5 | 0.651348141 | 7.163283115 | 7.438909576 | 7.20E-13 | 5.20E-12 | up |
| MCUB | 0.583964741 | 5.866574851 | 7.331051659 | 1.46E-12 | 9.94E-12 | up |
| EPCAM | 0.731158323 | 9.145808712 | 7.302925503 | 1.75E-12 | 1.18E-11 | up |
| CHI3L1 | 0.860429163 | 5.363176945 | 7.292915141 | 1.87E-12 | 1.25E-11 | up |
| COL10A1 | 0.776918601 | 4.331042955 | 7.271344675 | 2.15E-12 | 1.42E-11 | up |
| CD55 | 0.758115184 | 7.599265695 | 7.233281938 | 2.75E-12 | 1.81E-11 | up |
| CD14 | 0.605691212 | 8.071680097 | 7.125339617 | 5.49E-12 | 3.49E-11 | up |
| GUCY1A1 | 0.665063452 | 6.628579074 | 7.097203716 | 6.57E-12 | 4.10E-11 | up |
| VIL1 | 0.97439715 | 6.290091695 | 6.930734184 | 1.88E-11 | 1.08E-10 | up |
| IFI6 | 0.688983539 | 8.215676725 | 6.717449506 | 7.02E-11 | 3.68E-10 | up |
| PLA2G2A | 1.227132364 | 6.568024143 | 6.693153296 | 8.15E-11 | 4.22E-10 | up |
| VSNL1 | 0.687328639 | 4.26840539 | 6.651541435 | 1.05E-10 | 5.35E-10 | up |
| COL11A1 | 0.77248056 | 3.911101271 | 6.194810847 | 1.56E-09 | 6.69E-09 | up |
| SFRP2 | 1.015220656 | 6.987248977 | 5.63193671 | 3.54E-08 | 1.25E-07 | up |
| MUC13 | 0.873306362 | 7.845173134 | 5.567517814 | 4.98E-08 | 1.72E-07 | up |
| SLC19A3 | 0.683248846 | 4.257830988 | 5.531682022 | 6.02E-08 | 2.06E-07 | up |
| PTGS2 | 0.66000089 | 5.303918199 | 5.455044895 | 8.99E-08 | 3.02E-07 | up |
| CXCL11 | 0.624999338 | 4.608673591 | 5.179259571 | 3.67E-07 | 1.13E-06 | up |
| THBS4 | 0.735304524 | 5.724745601 | 5.039503545 | 7.33E-07 | 2.15E-06 | up |
| CCL20 | 0.695157849 | 6.288080321 | 4.422396691 | 1.29E-05 | 3.09E-05 | up |
| CXCL5 | 0.646519485 | 7.430233478 | 3.417883233 | 0.00070154 | 0.0013014 | up |
| REG4 | 0.671665313 | 6.402137746 | 3.070251269 | 0.00229731 | 0.0039534 | up |
| DGKD | -0.984041028 | 6.746981346 | -14.67153023 | 1.04E-38 | 2.35E-35 | down |
| MAL | -1.716761742 | 6.708061796 | -14.48107088 | 6.09E-38 | 9.18E-35 | down |
| ESRRG | -2.49613963 | 5.682517869 | -13.94451863 | 8.61E-36 | 6.48E-33 | down |
| ABCC5 | -0.882206194 | 6.532293648 | -13.57047812 | 2.63E-34 | 1.69E-31 | down |
| SST | -2.73123772 | 7.210182126 | -13.50752264 | 4.65E-34 | 2.63E-31 | down |
| MFSD4A | -2.341968286 | 6.80661409 | -13.18295286 | 8.78E-33 | 3.05E-30 | down |
| ERO1B | -1.458782777 | 5.063925588 | -13.07389117 | 2.34E-32 | 7.56E-30 | down |
| CAPN13 | -1.049682727 | 5.779822401 | -12.90349897 | 1.08E-31 | 3.25E-29 | down |
| GKN2 | -3.756315326 | 9.504064381 | -12.83706514 | 1.95E-31 | 5.19E-29 | down |
| CWH43 | -1.127323812 | 3.84515508 | -12.81986998 | 2.28E-31 | 5.71E-29 | down |
| GKN1 | -3.883899797 | 9.356745322 | -12.78689116 | 3.05E-31 | 7.26E-29 | down |
| SLC25A4 | -0.934621643 | 7.33265468 | -12.66661756 | 8.90E-31 | 1.93E-28 | down |
| GHRL | -2.245754483 | 6.305565109 | -12.6055823 | 1.53E-30 | 3.14E-28 | down |
| ADH7 | -1.366163007 | 4.124720405 | -12.51601853 | 3.38E-30 | 6.34E-28 | down |
| CEP85L | -0.788046665 | 5.028855032 | -12.51184296 | 3.51E-30 | 6.34E-28 | down |
| SLC26A9 | -1.634532169 | 5.807508735 | -12.3051447 | 2.17E-29 | 3.38E-27 | down |
| DPT | -1.391129505 | 6.484251348 | -12.09644104 | 1.35E-28 | 1.79E-26 | down |
| GLUL | -0.867277188 | 7.847234788 | -11.91000556 | 6.80E-28 | 8.11E-26 | down |
| GCNT2 | -0.905310979 | 5.003882944 | -11.90964051 | 6.82E-28 | 8.11E-26 | down |
| CAPN9 | -1.630358414 | 6.10668701 | -11.89270724 | 7.90E-28 | 9.15E-26 | down |
| PPP2R3A | -0.879366484 | 5.294505954 | -11.88139065 | 8.71E-28 | 9.84E-26 | down |
| CKMT2 | -1.429611152 | 5.242001398 | -11.84147233 | 1.23E-27 | 1.32E-25 | down |
| GPX3 | -1.044366771 | 7.424107892 | -11.82086773 | 1.47E-27 | 1.51E-25 | down |
| KIT | -1.13514871 | 6.126043109 | -11.79276958 | 1.87E-27 | 1.88E-25 | down |
| HPGD | -1.732525328 | 7.56226764 | -11.7740591 | 2.20E-27 | 2.12E-25 | down |
| STX12 | -0.838588384 | 7.759619407 | -11.76623991 | 2.35E-27 | 2.22E-25 | down |
| SULT1B1 | -1.359606581 | 6.199711982 | -11.69754674 | 4.25E-27 | 3.69E-25 | down |
| ARHGEF37 | -0.830446707 | 5.652970176 | -11.66524481 | 5.61E-27 | 4.69E-25 | down |
| TENT5C | -1.156714715 | 7.962898059 | -11.65767567 | 5.99E-27 | 4.90E-25 | down |
| CHIA | -2.872141793 | 5.766868837 | -11.65611496 | 6.07E-27 | 4.90E-25 | down |
| ARHGEF28 | -0.74793203 | 5.474407516 | -11.62485913 | 7.93E-27 | 6.29E-25 | down |
| APOBEC2 | -1.14298598 | 5.047062218 | -11.59527157 | 1.02E-26 | 7.83E-25 | down |
| FAM107A | -0.89887127 | 6.481905307 | -11.53540774 | 1.70E-26 | 1.26E-24 | down |
| CYSTM1 | -1.172482971 | 10.17767469 | -11.48589991 | 2.60E-26 | 1.90E-24 | down |
| GC | -1.649239745 | 4.724480599 | -11.40894443 | 5.01E-26 | 3.59E-24 | down |
| HADH | -0.585749768 | 8.063639183 | -11.40677358 | 5.10E-26 | 3.60E-24 | down |
| SMIM14 | -0.707324213 | 7.730213636 | -11.39257371 | 5.75E-26 | 3.94E-24 | down |
| SPTSSB | -0.8187735 | 4.516992613 | -11.35840633 | 7.69E-26 | 5.11E-24 | down |
| ADTRP | -1.654820585 | 6.734456208 | -11.33292918 | 9.54E-26 | 6.25E-24 | down |
| FAM13A | -0.736627799 | 6.578493566 | -11.30762489 | 1.18E-25 | 7.63E-24 | down |
| COBLL1 | -0.823346783 | 7.071902132 | -11.30369361 | 1.22E-25 | 7.78E-24 | down |
| FMO5 | -1.181303933 | 6.210342865 | -11.29112365 | 1.36E-25 | 8.53E-24 | down |
| BMP6 | -0.586424608 | 5.060286822 | -11.26663132 | 1.67E-25 | 1.03E-23 | down |
| KLF15 | -0.847088402 | 5.055742182 | -11.2587709 | 1.79E-25 | 1.09E-23 | down |
| ETFDH | -0.703618341 | 6.068970762 | -11.25774726 | 1.80E-25 | 1.09E-23 | down |
| AMPD1 | -0.984749791 | 4.217489309 | -11.24052804 | 2.08E-25 | 1.24E-23 | down |
| ELL2 | -0.816967384 | 7.175380412 | -11.23074193 | 2.26E-25 | 1.33E-23 | down |
| FGA | -1.641573074 | 5.030817431 | -11.19969903 | 2.94E-25 | 1.66E-23 | down |
| LIFR | -1.387152251 | 5.66749928 | -11.16009327 | 4.10E-25 | 2.21E-23 | down |
| PM20D1 | -0.643713476 | 3.742394837 | -11.12072331 | 5.71E-25 | 3.00E-23 | down |
| SLC9A2 | -1.138744535 | 5.5384237 | -10.99455982 | 1.64E-24 | 8.15E-23 | down |
| KCNJ13 | -0.754327784 | 3.34187371 | -10.95479545 | 2.29E-24 | 1.10E-22 | down |
| LYPD6B | -1.187475573 | 6.312788512 | -10.88808676 | 3.98E-24 | 1.82E-22 | down |
| FUT9 | -1.807926838 | 4.952037825 | -10.82195618 | 6.89E-24 | 3.03E-22 | down |
| CYFIP2 | -0.858916826 | 6.825804302 | -10.74132669 | 1.34E-23 | 5.72E-22 | down |
| AADAC | -1.489756132 | 5.918444259 | -10.73370981 | 1.43E-23 | 6.04E-22 | down |
| TFCP2L1 | -0.928075703 | 6.308193815 | -10.72870374 | 1.49E-23 | 6.23E-22 | down |
| RASSF6 | -0.879632919 | 5.673709005 | -10.67968866 | 2.23E-23 | 9.25E-22 | down |
| CYB5R1 | -0.71797983 | 7.239849961 | -10.66430835 | 2.53E-23 | 1.03E-21 | down |
| ANXA10 | -2.19884462 | 8.460211125 | -10.64288032 | 3.02E-23 | 1.20E-21 | down |
| DNER | -1.287316562 | 4.542556845 | -10.57522883 | 5.26E-23 | 2.02E-21 | down |
| GPR155 | -1.515149444 | 5.740848691 | -10.56896999 | 5.53E-23 | 2.08E-21 | down |
| GSTA1 | -1.833071331 | 6.669707356 | -10.52819074 | 7.72E-23 | 2.86E-21 | down |
| SELENBP1 | -0.93922355 | 8.045087049 | -10.52398783 | 7.99E-23 | 2.94E-21 | down |
| ADH1A | -1.023555361 | 5.017896582 | -10.51159878 | 8.84E-23 | 3.20E-21 | down |
| HTR1E | -0.594204712 | 3.711253661 | -10.43829007 | 1.61E-22 | 5.59E-21 | down |
| ADRB2 | -0.646507455 | 5.310519427 | -10.38119297 | 2.55E-22 | 8.68E-21 | down |
| NR3C2 | -0.685518689 | 5.698373444 | -10.36683184 | 2.87E-22 | 9.68E-21 | down |
| ACADL | -0.715590917 | 3.567291812 | -10.29088263 | 5.30E-22 | 1.74E-20 | down |
| SLC22A23 | -0.653542598 | 6.90469037 | -10.27643347 | 5.96E-22 | 1.92E-20 | down |
| PGC | -2.413102412 | 11.39063565 | -10.20078883 | 1.10E-21 | 3.46E-20 | down |
| GPAT3 | -0.791161798 | 5.937584684 | -10.16329158 | 1.48E-21 | 4.52E-20 | down |
| MUCL3 | -2.190953458 | 7.253961906 | -10.16166389 | 1.50E-21 | 4.55E-20 | down |
| EPB41L4A | -0.640026453 | 5.061587682 | -10.15435872 | 1.59E-21 | 4.76E-20 | down |
| SLC9A1 | -0.734428954 | 7.288709642 | -10.15242428 | 1.61E-21 | 4.80E-20 | down |
| KIAA1324 | -1.575530815 | 8.016113008 | -10.15019163 | 1.64E-21 | 4.85E-20 | down |
| SORBS2 | -0.79685958 | 6.20020902 | -10.14945015 | 1.65E-21 | 4.85E-20 | down |
| IQGAP2 | -0.79090773 | 7.317466404 | -10.14177822 | 1.76E-21 | 5.12E-20 | down |
| JCHAIN | -1.687070635 | 10.59185976 | -10.06313703 | 3.29E-21 | 9.36E-20 | down |
| PRDM16 | -0.75789862 | 6.021503923 | -9.987084341 | 6.03E-21 | 1.62E-19 | down |
| ARHGAP24 | -0.657605255 | 5.994320532 | -9.934697475 | 9.14E-21 | 2.35E-19 | down |
| ZNF385B | -0.933970952 | 4.272256765 | -9.855260588 | 1.71E-20 | 4.16E-19 | down |
| OXCT1 | -0.805822157 | 6.050538511 | -9.852756101 | 1.74E-20 | 4.22E-19 | down |
| ETNPPL | -1.412914909 | 3.701091163 | -9.781002981 | 3.07E-20 | 7.15E-19 | down |
| GNMT | -0.764551919 | 4.445396463 | -9.709988504 | 5.35E-20 | 1.20E-18 | down |
| TMEM161B | -0.806047126 | 6.239011328 | -9.692564234 | 6.13E-20 | 1.36E-18 | down |
| CPB1 | -0.79209986 | 3.73572138 | -9.682134196 | 6.65E-20 | 1.47E-18 | down |
| MYRIP | -0.970394758 | 4.624005861 | -9.65336517 | 8.32E-20 | 1.82E-18 | down |
| MYOC | -0.927890089 | 4.509150256 | -9.65145871 | 8.44E-20 | 1.83E-18 | down |
| NR0B2 | -0.851143248 | 5.729001498 | -9.649410094 | 8.58E-20 | 1.85E-18 | down |
| VILL | -0.831008182 | 6.927959579 | -9.628398606 | 1.01E-19 | 2.15E-18 | down |
| PRKACB | -0.803070528 | 6.661717305 | -9.62596687 | 1.03E-19 | 2.18E-18 | down |
| ALKAL2 | -0.892842874 | 3.67926809 | -9.583485458 | 1.43E-19 | 2.89E-18 | down |
| PER3 | -0.70940396 | 6.086125267 | -9.572054555 | 1.56E-19 | 3.14E-18 | down |
| BTG2 | -0.636092046 | 8.062919324 | -9.549372068 | 1.86E-19 | 3.68E-18 | down |
| TMEM171 | -0.866771504 | 6.351056977 | -9.494084566 | 2.86E-19 | 5.45E-18 | down |
| RPRM | -0.807486691 | 4.797413299 | -9.483883536 | 3.09E-19 | 5.85E-18 | down |
| RAP1GAP | -1.059793666 | 6.653999966 | -9.435495105 | 4.49E-19 | 8.18E-18 | down |
| ECI2 | -0.742922861 | 6.804122658 | -9.423969118 | 4.90E-19 | 8.79E-18 | down |
| SYTL1 | -0.608049916 | 6.271991565 | -9.401247563 | 5.84E-19 | 1.03E-17 | down |
| ATP13A4 | -0.587627982 | 3.95638219 | -9.323768789 | 1.06E-18 | 1.78E-17 | down |
| SULT1C2 | -1.515644521 | 7.727853453 | -9.284279768 | 1.43E-18 | 2.32E-17 | down |
| RORC | -0.630081828 | 5.68015673 | -9.239182629 | 2.01E-18 | 3.22E-17 | down |
| SH3RF2 | -0.685003375 | 6.286844935 | -9.201163567 | 2.68E-18 | 4.22E-17 | down |
| HHIP | -0.76640508 | 5.130931123 | -9.157072073 | 3.74E-18 | 5.73E-17 | down |
| GSTA4 | -0.692334225 | 6.809191767 | -9.105556745 | 5.51E-18 | 8.20E-17 | down |
| TENT5A | -0.587912884 | 7.9151254 | -9.070347653 | 7.18E-18 | 1.05E-16 | down |
| CCKAR | -1.202286752 | 5.416154365 | -9.0677665 | 7.32E-18 | 1.06E-16 | down |
| SH3BGRL2 | -0.704914326 | 7.900155649 | -9.038098502 | 9.15E-18 | 1.31E-16 | down |
| NNT | -0.65562247 | 7.37503267 | -9.016517756 | 1.07E-17 | 1.53E-16 | down |
| IGFBP2 | -1.069259148 | 8.455852142 | -9.002500004 | 1.19E-17 | 1.69E-16 | down |
| LTF | -1.720956888 | 7.628152253 | -8.983413362 | 1.38E-17 | 1.91E-16 | down |
| BVES | -0.734577236 | 4.78911125 | -8.970585204 | 1.51E-17 | 2.09E-16 | down |
| GUCA2B | -0.823584358 | 4.37469551 | -8.947890755 | 1.79E-17 | 2.44E-16 | down |
| SUCLG2 | -0.674477517 | 7.967098929 | -8.8574322 | 3.51E-17 | 4.66E-16 | down |
| GHR | -0.80009208 | 3.974177099 | -8.853072014 | 3.62E-17 | 4.79E-16 | down |
| HYAL1 | -0.775206509 | 6.578085639 | -8.835988132 | 4.11E-17 | 5.39E-16 | down |
| MLPH | -0.819011753 | 8.092687708 | -8.656938198 | 1.53E-16 | 1.88E-15 | down |
| C1orf116 | -0.782514862 | 7.381309357 | -8.626145751 | 1.91E-16 | 2.33E-15 | down |
| NOSTRIN | -0.683198428 | 5.900827907 | -8.616919259 | 2.05E-16 | 2.48E-15 | down |
| MZB1 | -0.912271789 | 6.710940161 | -8.579924138 | 2.68E-16 | 3.20E-15 | down |
| RCAN2 | -0.662641499 | 7.009688661 | -8.536464322 | 3.66E-16 | 4.29E-15 | down |
| RBM47 | -0.582043705 | 8.271145826 | -8.536407331 | 3.67E-16 | 4.29E-15 | down |
| LAMA2 | -0.650885434 | 5.775268723 | -8.465103612 | 6.13E-16 | 7.01E-15 | down |
| CLDN18 | -1.545773531 | 9.966909383 | -8.422251754 | 8.33E-16 | 9.32E-15 | down |
| TPD52L1 | -0.874567673 | 6.581304129 | -8.366876101 | 1.24E-15 | 1.35E-14 | down |
| ALCAM | -0.59385488 | 7.135166849 | -8.302981248 | 1.95E-15 | 2.05E-14 | down |
| GREM2 | -0.746879812 | 5.435799283 | -8.301951282 | 1.96E-15 | 2.06E-14 | down |
| MRAP2 | -0.815852075 | 5.655203332 | -8.270634225 | 2.45E-15 | 2.55E-14 | down |
| PROM2 | -0.775813184 | 6.768453239 | -8.23600726 | 3.13E-15 | 3.21E-14 | down |
| PKIB | -0.784975272 | 5.384718127 | -8.179405995 | 4.66E-15 | 4.68E-14 | down |
| C6orf58 | -2.104574457 | 6.524866227 | -8.173470046 | 4.86E-15 | 4.84E-14 | down |
| ATP8A1 | -0.666557856 | 5.883788569 | -8.114812011 | 7.33E-15 | 7.19E-14 | down |
| P2RY14 | -0.683114644 | 5.646654933 | -8.098830992 | 8.20E-15 | 7.91E-14 | down |
| ADH1B | -1.203853914 | 5.661390429 | -8.070107881 | 1.00E-14 | 9.52E-14 | down |
| TCEA3 | -0.742241969 | 7.658317229 | -8.04335321 | 1.21E-14 | 1.12E-13 | down |
| CYP4X1 | -0.960693451 | 5.347598927 | -7.927559855 | 2.68E-14 | 2.39E-13 | down |
| ANGPTL3 | -0.625733114 | 2.614912131 | -7.726055046 | 1.06E-13 | 8.70E-13 | down |
| POPDC3 | -0.742728057 | 3.832357669 | -7.616818577 | 2.21E-13 | 1.74E-12 | down |
| NECTIN3 | -0.626226892 | 6.243247825 | -7.528991845 | 3.97E-13 | 2.99E-12 | down |
| REG1A | -1.712274171 | 9.905507222 | -7.520196602 | 4.21E-13 | 3.16E-12 | down |
| P3H2 | -0.600344438 | 6.016048907 | -7.483796012 | 5.35E-13 | 3.97E-12 | down |
| SLC4A4 | -0.881570685 | 6.23535432 | -7.463277149 | 6.13E-13 | 4.50E-12 | down |
| MECOM | -0.645261384 | 7.9733625 | -7.426465554 | 7.81E-13 | 5.61E-12 | down |
| ARL14 | -0.944427253 | 6.028076741 | -7.349479662 | 1.29E-12 | 8.87E-12 | down |
| CSTA | -0.749211303 | 5.806313494 | -7.327649715 | 1.49E-12 | 1.01E-11 | down |
| STX19 | -0.66102761 | 4.404753421 | -7.175730288 | 3.98E-12 | 2.58E-11 | down |
| SERPINI1 | -0.598842237 | 4.477850411 | -7.131731547 | 5.27E-12 | 3.36E-11 | down |
| DDX60 | -0.698241561 | 6.533461911 | -7.078293497 | 7.41E-12 | 4.59E-11 | down |
| ENPP5 | -0.658095037 | 3.452299248 | -6.996050355 | 1.25E-11 | 7.44E-11 | down |
| PI16 | -0.587471933 | 6.021122732 | -6.984855856 | 1.34E-11 | 7.96E-11 | down |
| CCL28 | -0.707016877 | 5.619458724 | -6.946990338 | 1.70E-11 | 9.94E-11 | down |
| PTGER3 | -0.702328513 | 3.732491232 | -6.824975102 | 3.63E-11 | 2.00E-10 | down |
| FMOD | -0.633919211 | 7.691390757 | -6.690419301 | 8.28E-11 | 4.27E-10 | down |
| SELENOP | -0.623276913 | 8.158735839 | -6.58250631 | 1.59E-10 | 7.86E-10 | down |
| CPE | -0.602301169 | 5.967048566 | -6.486379645 | 2.83E-10 | 1.33E-09 | down |
| S100P | -0.831544821 | 9.835654384 | -6.376300014 | 5.43E-10 | 2.48E-09 | down |
| LRRC31 | -0.619583589 | 4.807826144 | -6.368287566 | 5.69E-10 | 2.59E-09 | down |
| SPINK1 | -1.055990793 | 10.32284264 | -6.117310467 | 2.43E-09 | 1.01E-08 | down |
| ECRG4 | -0.843372103 | 6.320904007 | -6.088427239 | 2.86E-09 | 1.17E-08 | down |
| IRX2 | -0.591572696 | 6.327031893 | -6.050526395 | 3.55E-09 | 1.43E-08 | down |
| HMGCS2 | -0.913815958 | 7.672451311 | -6.034044604 | 3.89E-09 | 1.56E-08 | down |
| IL1R2 | -0.734237434 | 6.889932447 | -6.031697918 | 3.95E-09 | 1.58E-08 | down |
| REG3A | -1.312986041 | 6.562576949 | -5.897713239 | 8.34E-09 | 3.23E-08 | down |
| CXCL14 | -0.669059085 | 7.82679968 | -5.746269826 | 1.91E-08 | 6.97E-08 | down |
| F13A1 | -0.674981245 | 6.614234145 | -5.684603632 | 2.67E-08 | 9.53E-08 | down |
| LIPH | -0.620291824 | 6.876469257 | -5.608414594 | 4.01E-08 | 1.41E-07 | down |
| PIGR | -1.019644409 | 9.976962961 | -5.544053503 | 5.64E-08 | 1.94E-07 | down |
| FGG | -0.69629576 | 3.055475512 | -5.321757339 | 1.79E-07 | 5.79E-07 | down |
| MUC1 | -0.590094637 | 9.562736027 | -5.289776338 | 2.10E-07 | 6.75E-07 | down |
| ODAM | -0.580543722 | 4.425862181 | -5.269708187 | 2.33E-07 | 7.44E-07 | down |
| CPA3 | -0.612519904 | 6.503396019 | -5.260803579 | 2.44E-07 | 7.74E-07 | down |
| PLAC8 | -0.74590986 | 8.750963251 | -5.190751487 | 3.47E-07 | 1.08E-06 | down |
| FAM3D | -0.686200589 | 7.357622722 | -5.163513983 | 3.97E-07 | 1.22E-06 | down |
| UPK1B | -0.583676041 | 5.061495162 | -4.879442125 | 1.59E-06 | 4.41E-06 | down |
| TSPAN1 | -0.646204499 | 8.595553991 | -4.807610063 | 2.23E-06 | 6.02E-06 | down |
| RBP2 | -0.58235743 | 5.167448385 | -3.592665747 | 0.0003717 | 0.0007206 | down |
| CLCA1 | -0.793759461 | 4.850679573 | -3.514447771 | 0.00049549 | 0.0009412 | down |

**Supplemental Table 2:** Significant models were obtained from the PPI network based on the MCODE analysis in Cytoscape.

| Module | gene | | logFC | P(HR)  GEPIA | | logrank P GEPIA | logrank P KAPLAN MEIER PLOTTER |
| --- | --- | --- | --- | --- | --- | --- | --- |
| Module1;MCODE score=26.148 | | STIL | 0.883187843 | 0.11 | 0.11 | | 0.0049 |
|  |  | NUF2 | 0.998163722 | 0.97 | 0.98 | | 8.30E-06 |
|  |  | BUB1 | 0.998373094 | 0.77 | 0.77 | | 7.80E-05 |
|  |  | DEPDC1 | 0.767577941 | 0.58 | 0.58 | | 5.70E-09 |
|  |  | KIF2C | 0.78547097 | 0.5 | 0.5 | | 0.08 |
|  |  | NEK2 | 0.741768032 | 0.6 | 0.6 | | 3.90E-07 |
|  |  | CDCA7 | 0.900934121 | 0.11 | 0.11 | | 0.0035 |
|  |  | TRIP13 | 1.020903833 | 0.99 | 0.99 | | 0.013 |
|  |  | RRM2 | 1.053497706 | 0.26 | 0.26 | | 1.00E-06 |
|  |  | SPC25 | 0.866017259 | 0.23 | 0.23 | | 7.70E-06 |
|  |  | MAD2L1 | 0.747451624 | 0.63 | 0.63 | | 3.30E-09 |
|  |  | CDC20 | 1.058805839 | 0.8 | 0.81 | | 0.37 |
|  |  | ECT2 | 1.187573744 | 0.011 | 0.01 | | 0.37 |
|  |  | CCNA2 | 0.802525506 | 0.6 | 0.6 | | 3.90E-07 |
|  |  | EXO1 | 0.755240932 | 0.6 | 0.61 | | 0.13 |
|  |  | CENPK | 0.692109397 | 0.41 | 0.42 | | 2.60E-05 |
|  |  | KIF15 | 0.643823447 | 0.9 | 0.9 | | 0.014 |
|  |  | KIF14 | 0.674392981 | 0.56 | 0.56 | | 0.24 |
|  |  | TTK | 0.745533237 | 0.6 | 0.61 | | 0.0015 |
|  |  | PTTG1 | 0.934500054 | 0.86 | 0.86 | | 9.70E-05 |
|  |  | DTL | 0.836441461 | 0.47 | 0.47 | | 0.0013 |
|  |  | UBE2T | 1.041371364 | 0.11 | 0.11 | | 1.10E-05 |
|  |  | HMMR | 0.662987341 | 0.31 | 0.31 | | 4.40E-09 |
|  |  | ASPM | 0.835044994 | 0.23 | 0.23 | | 1.40E-06 |
|  |  | CCNB1 | 1.032618483 | 0.65 | 0.66 | | 8.60E-07 |
|  |  | DEPDC1B | 0.808856028 | 0.55 | 0.54 | | 4.80E-07 |
|  |  | SMC4 | 0.613524606 | 0.26 | 0.26 | | 0.16 |
|  |  | CENPF | 0.969981978 | 0.29 | 0.29 | | 0.059 |
| Module2;MCODE score=12 | | HTR1E | -0.594204712 |  |  | | 0.00011 |
|  |  | PTGER3 | -0.702328513 | 0.3 | 0.3 | | 7.60E-06 |
|  |  | AGT | 0.734330077 | 0.02 | 0.02 | | 0.0017 |
|  |  | CCL20 | 0.695157849 | 0.92 | 0.93 | | 2.30E-07 |
|  |  | SST | -2.73123772 | 0.2 | 0.2 | | 0.046 |
|  |  | CXCL8 | 1.547177115 | 1 | 1 | | 1.40E-05 |
|  |  | CXCL9 | 0.984634472 | 0.49 | 0.49 | | 9.80E-09 |
|  |  | CXCL10 | 1.095641709 | 0.93 | 0.93 | | 1.00E-07 |
|  |  | CXCL11 | 0.624999338 | 0.37 | 0.37 | | 1.30E-09 |
|  |  | CCL28 | -0.707016877 | 0.46 | 0.46 | | 0.0017 |
|  |  | P2RY14 | -0.683114644 | 0.032 | 0.032 | | 0.0016 |
|  |  | CXCL5 | 0.646519485 | 0.79 | 0.78 | | 1.80E-05 |
| Module3;MCODE score=4.800 | | HMGCS2 | -0.913815958 | 0.36 | 0.36 | | 0.012 |
|  |  | ACADL | -0.715590917 | 0.022 | 0.022 | | 2.30E-06 |
|  |  | HADH | -0.585749768 | 0.96 | 0.97 | | 2.30E-08 |
|  |  | OXCT1 | -0.805822157 | 0.5 | 0.5 | | 0.00088 |
|  |  | ETFDH | -0.703618341 | 0.93 | 0.93 | | 1.50E-07 |
|  |  | ECI2 | -0.742922861 | 0.25 | 0.25 | |  |
| Module4;MCODE score=4.000 | | COL3A1 | 1.079522626 | 0.12 | 0.12 | | 0.069 |
|  |  | IGFBP7 | 0.672618901 | 0.0045 | 0.0043 | | 2.30E-05 |
|  |  | PDGFRB | 0.764474822 | 0.026 | 0.025 | | 9.70E-12 |
|  |  | PTGS2 | 0.66000089 | 0.033 | 0.032 | | 0.0013 |
|  |  | FN1 | 1.23674502 | 0.051 | 0.05 | | 1.30E-05 |
|  |  | FGA | -1.641573074 | 0.66 | 0.67 | | 0.00075 |
|  |  | P3H2 | -0.600344438 | 0.14 | 0.14 | | 0.0083 |
|  |  | LAMA2 | -0.650885434 | 0.023 | 0.023 | | 1.40E-05 |
|  |  | FGG | -0.69629576 | 0.043 | 0.042 | | 1.40E-05 |
|  |  | COL10A1 | 0.776918601 | 0.083 | 0.084 | | 0.011 |
|  |  | FMOD | -0.633919211 | 0.037 | 0.036 | | 0.00052 |
| Module5;MCODE score=4.000 | | ADH1A | -1.023555361 | 0.46 | 0.45 | | 0.17 |
|  |  | GSTA4 | -0.692334225 | 0.24 | 0.24 | | 0.00029 |
|  |  | ADH1B | -1.203853914 | 0.0073 | 0.0068 | | 0.041 |
|  |  | GSTA1 | -1.833071331 | 0.095 | 0.094 | | 0.02 |
| Module6;MCODE score=3.000 | | F2R | 0.756760839 | 0.015 | 0.015 | | 0.13 |
|  |  | F2RL2 | 0.817066044 | 0.51 | 0.52 | | 2.20E-06 |
|  |  | EDNRA | 0.749100877 | 0.016 | 0.015 | | 7.70E-06 |
| Module7;MCODE score=3.000 | | SPARC | 1.256562042 | 0.052 | 0.051 | | 0.009 |
|  |  | COL5A2 | 1.027501146 | 0.01 | 0.0095 | | 0.19 |
|  |  | COL6A3 | 1.282522225 | 0.22 | 0.21 | | 0.0018 |
| Module8;MCODE score=3.000 | | SLC4A4 | -0.881570685 | 0.97 | 0.97 | | 1.80E-06 |
|  |  | SLC9A2 | -1.138744535 | 0.78 | 0.78 | | 5.00E-07 |
|  |  | SLC26A9 | -1.634532169 | 0.86 | 0.86 | | 0.046 |
| Module9;MCODE score=2.667 | | SPP1 | 2.130579253 | 0.65 | 0.67 | | 2.70E-14 |
|  |  | VCAN | 0.933460244 | 0.0016 | 0.0014 | | 0.0033 |
|  |  | ITGA2 | 0.997780752 | 0.74 | 0.73 | | 0.0039 |
|  |  | COL11A1 | 0.77248056 | 0.87 | 0.86 | | 0.018 |

**Supplemental Table 3:** The determined selected genes by using the cytoHubba plugin such as degree, betweenness centrality, and closeness.

| genes name | Betweenness | Closeness | Degree |
| --- | --- | --- | --- |
| CDC20 | 216.21093 | 92.90238 | 33 |
| NEK2 | 843.54131 | 94.71667 | 28 |
| BUB1 | 191.40557 | 92.40238 | 32 |
| MAD2L1 | 2936.2939 | 94.20238 | 33 |
| NUF2 | 96.97332 | 90.60238 | 30 |
| SPC25 | 6.86061 | 85.21905 | 24 |
| CCNA2 | 1835.44704 | 101.26667 | 36 |
| PTTG1 | 157.44364 | 90.90238 | 29 |
| TRIP13 | 1.93954 | 85.71905 | 25 |
| CCNB1 | 5158.47402 | 105.31667 | 38 |
| CTNNB1 | 7342.39478 | 105.51667 | 24 |
| LEF1 | 149.57858 | 81.35 | 5 |
| SKP2 | 2019.29416 | 81.74286 | 14 |
| FGG | 945.10316 | 88.01667 | 13 |
| FGA | 693.23674 | 86.6 | 12 |
| TTK | 93.29951 | 90.60238 | 30 |
| CENPF | 70.87458 | 90.43571 | 30 |
| KIF2C | 697.33641 | 90.31905 | 32 |
| ASPM | 70.87458 | 90.43571 | 30 |
| COL5A2 | 303.21729 | 84.85 | 15 |
| COL3A1 | 1150.84646 | 91.73333 | 24 |
| KIF15 | 462.26547 | 88.38571 | 30 |
| CCL20 | 1141.15212 | 90.46667 | 18 |
| CXCL8 | 5911.57275 | 106.3 | 33 |
| CXCL11 | 219.92994 | 84 | 14 |
| CXCL10 | 1672.86893 | 95.75 | 22 |
| CXCL9 | 131.97152 | 81.25238 | 13 |
| E2F3 | 155.16274 | 81.81667 | 8 |
| COL6A3 | 176.75432 | 81.15238 | 14 |
| CD55 | 1063.49017 | 74.20238 | 6 |
| C2 | 0 | 54.5881 | 1 |
| HMMR | 4570.36901 | 101.31667 | 34 |
| FN1 | 7818.60296 | 110.96667 | 41 |
| SPARC | 414.92273 | 88.38333 | 18 |
| ADH1B | 0 | 53.88333 | 3 |
| ADH1A | 905.99327 | 66.52143 | 5 |
| CENPK | 123.63182 | 85.10238 | 23 |
| CXCL5 | 85.97651 | 86.5 | 14 |
| SPP1 | 3272.95776 | 102.76667 | 28 |
| RUVBL1 | 1032.17642 | 84.41667 | 8 |
| SMC4 | 934.40908 | 88.76905 | 27 |
| F13A1 | 949.9254 | 77.48571 | 7 |
| VCAN | 3189.96302 | 97.83333 | 18 |
| MUC13 | 748.27739 | 73.66667 | 5 |
| MUC1 | 547.3566 | 87.71667 | 8 |
| COL12A1 | 174.83773 | 80.58571 | 13 |
| RRM2 | 1024.70588 | 93.31905 | 33 |
| HMGCS2 | 142.1972 | 53.41667 | 5 |
| OXCT1 | 10.19048 | 46.92341 | 4 |
| ITGA2 | 1257.00375 | 88.23333 | 14 |
| LMNB1 | 493.3423 | 88.25 | 18 |
| MYB | 757.17266 | 85.26667 | 7 |
| ECT2 | 454.08555 | 87.38571 | 28 |
| PTGS2 | 5694.52019 | 103.63333 | 20 |
| AGT | 3335.47942 | 99.9 | 28 |
| EDNRA | 422.55785 | 87.11667 | 10 |
| HADH | 143.77849 | 56.88571 | 5 |
| STMN1 | 434.02346 | 82.9 | 10 |
| KIF2A | 13.51566 | 77.20238 | 14 |
| STIL | 0 | 82.88571 | 20 |
| COL11A1 | 571.147 | 83.68333 | 13 |
| MFAP2 | 448 | 76.41667 | 4 |
| LOX | 999.11359 | 96.01667 | 21 |
| F2R | 2133.35765 | 84.43571 | 11 |
| F2RL2 | 218.7071 | 80.78333 | 6 |
| DTL | 65.89426 | 88.35238 | 28 |
| EXO1 | 59.51574 | 89.93571 | 29 |
| DEPDC1 | 3.44094 | 86.38571 | 26 |
| LAMC2 | 0 | 62.36905 | 2 |
| PTGER3 | 182.97332 | 85.75 | 14 |
| ADRB2 | 138.62307 | 78.36667 | 5 |
| IGFBP7 | 310.54633 | 85.6 | 10 |
| KIF14 | 18.2381 | 86.38571 | 26 |
| CCL28 | 52.40623 | 80.48571 | 13 |
| SST | 601.66471 | 87.75 | 17 |
| COL10A1 | 1101.18832 | 81.26667 | 10 |
| CHI3L1 | 1143.21132 | 77.38571 | 6 |
| LTF | 501.6389 | 74.8 | 6 |
| LAMA2 | 105.28672 | 77.26905 | 9 |
| PRKACB | 2529.54912 | 95.16667 | 10 |
| IQGAP2 | 461.34545 | 71.33333 | 3 |
| CPB1 | 1752.17616 | 80.06667 | 7 |
| GCNT2 | 0 | 46.2131 | 1 |
| FUT9 | 448 | 58.81667 | 2 |
| TUBB | 649.64446 | 78.91905 | 11 |
| PVRL3 | 710.60101 | 64.93571 | 3 |
| CLDN1 | 679.0753 | 65.15 | 3 |
| SERPINE2 | 883.24546 | 77.35238 | 7 |
| P2RY14 | 0 | 78.73571 | 11 |
| LEPREL1 | 0 | 67.32143 | 6 |
| HTR1E | 34.97689 | 79.98571 | 12 |
| CLDN18 | 10.55714 | 51.22143 | 2 |
| CPA3 | 3.22864 | 70.03571 | 2 |
| TNFAIP6 | 1005.33361 | 92.18333 | 8 |
| LBR | 0 | 66.81905 | 2 |
| FCER1G | 212.56111 | 68.86905 | 5 |
| FCGR2A | 1880.22611 | 80.76667 | 10 |
| CD14 | 657.59092 | 83.65 | 9 |
| SLC9A1 | 2581.77198 | 93.41667 | 8 |
| GKN1 | 0 | 1 | 1 |
| CAPN9 | 0 | 1 | 1 |
| CCKAR | 63.42124 | 74.65238 | 7 |
| PIGR | 1162.02536 | 69.38333 | 5 |
| IGJ | 984.64704 | 60.91905 | 4 |
| ATP8A1 | 556.07846 | 60.74524 | 3 |
| CYSTM1 | 11.2 | 56.83571 | 2 |
| CYFIP2 | 489.43381 | 59.91667 | 2 |
| PLAC8 | 0 | 56.64048 | 1 |
| GC | 616.48288 | 73.66905 | 6 |
| RPF2 | 0 | 51.70952 | 1 |
| HEATR1 | 634.43065 | 68.95238 | 4 |
| PLA2G2A | 517.09167 | 76.88333 | 5 |
| PLA2G7 | 0 | 56.35714 | 1 |
| UBE2T | 0.60325 | 83.55238 | 21 |
| CDCA7 | 1.00368 | 83.55238 | 21 |
| GPX3 | 15.97569 | 48.60357 | 3 |
| GSTA4 | 514.14162 | 61.3619 | 6 |
| REG3A | 826.93023 | 76.08571 | 5 |
| REG1A | 82.21265 | 62.67143 | 3 |
| GPX8 | 1762.51113 | 72.38333 | 4 |
| TGFBI | 27.13311 | 76.51905 | 7 |
| CXCL14 | 0 | 72.98571 | 6 |
| GSTA1 | 605.6199 | 61.8619 | 7 |
| SEPP1 | 90.69463 | 54.70238 | 2 |
| SUCLG2 | 517.855 | 52.73056 | 4 |
| GHRL | 1439.48331 | 79.46905 | 12 |
| HPGD | 121.85418 | 76.05 | 4 |
| DEPDC1B | 448.3005 | 84.05238 | 22 |
| FMOD | 76.1351 | 82.55 | 10 |
| HHIP | 313.743 | 63.76905 | 2 |
| FAM13A | 548.2258 | 63.31667 | 3 |
| CPE | 14.68651 | 65.30476 | 2 |
| DDX60 | 451.89594 | 70.95 | 4 |
| IFI6 | 0 | 65.70238 | 2 |
| ETFDH | 2540.04373 | 68.24762 | 6 |
| ACADL | 850.80509 | 58.45238 | 7 |
| PER3 | 0 | 48.63333 | 1 |
| RORC | 448 | 62.9381 | 2 |
| MZB1 | 0 | 47.3 | 1 |
| SLC4A4 | 749.53538 | 67.46667 | 4 |
| ANGPTL3 | 460.56227 | 64.20476 | 4 |
| SELENBP1 | 542.94253 | 64.06667 | 3 |
| FAP | 137.24652 | 77.50238 | 6 |
| GLUL | 599.02053 | 79.2 | 5 |
| ADH7 | 0 | 47.9369 | 2 |
| THBS4 | 486.17688 | 77.23571 | 7 |
| GHR | 563.65984 | 75.50238 | 6 |
| ATP1B3 | 2031.59131 | 75.71667 | 5 |
| SYTL1 | 0 | 1 | 1 |
| MYRIP | 0 | 1 | 1 |
| EPCAM | 860.02896 | 85.18333 | 7 |
| CTSK | 0 | 75.91905 | 5 |
| SFRP2 | 0 | 70.33333 | 2 |
| PAICS | 3360.14285 | 88.68571 | 22 |
| CTPS1 | 87.45094 | 70.93571 | 4 |
| PDGFRB | 453.20188 | 88.43333 | 12 |
| ESRRG | 0 | 54.80476 | 1 |
| NR0B2 | 464.01628 | 73.7 | 4 |
| PRDM16 | 53.04004 | 64.07857 | 2 |
| REG4 | 57.95072 | 55.75714 | 2 |
| MYOC | 0 | 71.66905 | 2 |
| LAPTM5 | 0 | 58.58571 | 2 |
| HYAL1 | 0.66667 | 77.55 | 3 |
| STX12 | 0 | 1 | 1 |
| STX19 | 0 | 1 | 1 |
| ERO1LB | 0 | 49.62143 | 1 |
| SLC9A2 | 0 | 52.5619 | 2 |
| ALCAM | 177.66228 | 78.01905 | 7 |
| RPRM | 0 | 68.26905 | 1 |
| ODAM | 0 | 47.3 | 1 |
| ENO1 | 1471.77337 | 76.95 | 9 |
| KCNJ13 | 0 | 59.1381 | 1 |
| PGM2 | 714.55888 | 68.67857 | 4 |
| KIT | 575.44702 | 88.98333 | 10 |
| NNT | 231.22954 | 53.40238 | 2 |
| DPT | 240.25357 | 78.81905 | 6 |
| NR3C2 | 8.06825 | 68.21905 | 2 |
| ECI2 | 1948.83801 | 67.05238 | 7 |
| ARHGEF28 | 0 | 59.39762 | 1 |
| GREM2 | 57.94035 | 58.69762 | 2 |
| BMP6 | 460.49478 | 77.63333 | 5 |
| FAM107A | 0 | 1.5 | 1 |
| ETNPPL | 2 | 2 | 2 |
| ARL14 | 0 | 60.29762 | 1 |
| PGC | 374.84923 | 66.45 | 4 |
| VSNL1 | 586.22448 | 58.52143 | 3 |
| ANXA10 | 8 | 47.25556 | 2 |
| IGFBP2 | 33.66297 | 65.62143 | 4 |
| SLC26A9 | 60.33658 | 56.4381 | 3 |
| CLCA1 | 549.23921 | 71.95 | 3 |
| MFSD4 | 746.86302 | 72.50238 | 3 |
| RBP2 | 29.15133 | 70.35238 | 2 |
| SULT1C2 | 47.20009 | 58.12143 | 2 |
| SPINK1 | 128.32288 | 65.15476 | 3 |
| GUCY1A3 | 0 | 44.98413 | 1 |
| ADTRP | 448 | 56.8881 | 2 |
| ENPP5 | 448 | 48.36508 | 2 |
| RCAN2 | 1469.58827 | 61.7381 | 3 |
| GKN2 | 0 | 1 | 1 |
| RARRES1 | 0 | 1 | 1 |
| RASSF6 | 0 | 56.0381 | 1 |
| GUCA2B | 31.58089 | 60.20476 | 2 |
| IL1R2 | 0 | 71.48571 | 3 |
| PM20D1 | 0 | 1 | 1 |
| ATP13A4 | 0 | 1 | 1 |
| SMIM14 | 0 | 39.49921 | 1 |
| BTG2 | 0 | 57.84643 | 1 |
| PRRX1 | 241.09525 | 67.40476 | 4 |
| ZNF385B | 471.26008 | 50.69127 | 3 |
| ELOVL5 | 0 | 45.00675 | 1 |
| ANTXR1 | 0 | 60.29762 | 1 |
| DNER | 0 | 56.17143 | 1 |
| SERPINI1 | 495.39416 | 59.77143 | 3 |
| MECOM | 14.08482 | 55.43571 | 2 |
| SSB | 5.48085 | 68.06905 | 2 |
| BVES | 0 | 1 | 1 |
| POPDC3 | 0 | 1 | 1 |
| TMEM171 | 0 | 58.23095 | 1 |
| VIL1 | 75.53011 | 73.40238 | 3 |
| S100A10 | 169.44734 | 55.55476 | 2 |
| LYPD6B | 0 | 40.78254 | 1 |
| AGPAT9 | 0 | 41.28413 | 1 |
| ARHGAP24 | 448 | 51.39722 | 2 |
| RAP1GAP | 448 | 57.45476 | 2 |
| CKMT2 | 0 | 1.5 | 1 |
| PI16 | 0 | 46.71905 | 1 |
| DGKD | 0 | 53.95476 | 1 |
| SH3RF2 | 0 | 55.20714 | 1 |
| FAM3D | 0 | 47.34167 | 1 |
| ARHGEF37 | 0 | 45.23889 | 1 |
| SLC25A4 | 0 | 41.78413 | 1 |
| NT5DC2 | 44.03832 | 50.37857 | 2 |
| COBLL1 | 59.86196 | 50.44762 | 2 |
| KLF15 | 0 | 66.38571 | 1 |
| PKIB | 0 | 65.58333 | 1 |
| TFCP2L1 | 0 | 1 | 1 |
| LIFR | 0 | 1 | 1 |
| MYO1B | 0 | 53.55952 | 1 |
| OLFML2B | 0 | 62.35476 | 1 |
| PPP2R3A | 0 | 49.29048 | 1 |
| SLC22A23 | 0 | 50.33333 | 1 |
| KLHL23 | 0 | 59.73095 | 1 |
| RAI14 | 0 | 57.84643 | 1 |

**Supplemental Table 4 :** The determined selected genes of Venn diagram.

| Names | total | elements |
| --- | --- | --- |
| betweenness closeness degree | 7 | CCNB1 |
|  |  | CXCL8 |
|  |  | FN1 |
|  |  | MAD2L1 |
|  |  | SPP1 |
|  |  | HMMR |
|  |  | CCNA2 |
| betweenness closeness | 6 | AGT |
|  |  | CTNNB1 |
|  |  | VCAN |
|  |  | PRKACB |
|  |  | SLC9A1 |
|  |  | PTGS2 |
| closeness degree | 4 | RRM2 |
|  |  | BUB1 |
|  |  | CDC20 |
|  |  | NEK2 |
| betweenness | 7 | ECI2 |
|  |  | ETFDH |
|  |  | F2R |
|  |  | PAICS |
|  |  | SKP2 |
|  |  | FCGR2A |
|  |  | ATP1B3 |
| closeness | 3 | LOX |
|  |  | CXCL10 |
|  |  | TNFAIP6 |
| degree | 9 | NUF2 |
|  |  | EXO1 |
|  |  | CENPF |
|  |  | ASPM |
|  |  | ECT2 |
|  |  | TTK |
|  |  | PTTG1 |
|  |  | KIF15 |
|  |  | KIF2C |

**Supplemental Table 5 :** The gene-TF regulatory network was constructed including 129 interaction pairs among 7 genes and 102 TFs

| genes | connection | Transcriptional factors |
| --- | --- | --- |
| SPP1 | interacts with | SMAD4 |
| SPP1 | interacts with | CEBPA |
| SPP1 | interacts with | FOXJ2 |
| SPP1 | interacts with | CEBPG |
| SPP1 | interacts with | KLF16 |
| MAD2L1 | interacts with | RFX1 |
| MAD2L1 | interacts with | ZNF382 |
| MAD2L1 | interacts with | TBX21 |
| MAD2L1 | interacts with | TFDP1 |
| MAD2L1 | interacts with | IKZF1 |
| MAD2L1 | interacts with | E2F4 |
| MAD2L1 | interacts with | PRDM1 |
| MAD2L1 | interacts with | ZNF2 |
| MAD2L1 | interacts with | FOXA3 |
| MAD2L1 | interacts with | IRF1 |
| MAD2L1 | interacts with | BCL6 |
| MAD2L1 | interacts with | MLLT1 |
| MAD2L1 | interacts with | ETV4 |
| MAD2L1 | interacts with | RFXANK |
| MAD2L1 | interacts with | ZBTB11 |
| HMMR | interacts with | ZNF2 |
| HMMR | interacts with | ID3 |
| HMMR | interacts with | SP1 |
| HMMR | interacts with | ADNP |
| HMMR | interacts with | ZNF335 |
| HMMR | interacts with | NFRKB |
| HMMR | interacts with | JUNB |
| HMMR | interacts with | REST |
| HMMR | interacts with | CHD1 |
| HMMR | interacts with | PHF8 |
| HMMR | interacts with | INSM2 |
| HMMR | interacts with | HBP1 |
| HMMR | interacts with | KDM5B |
| HMMR | interacts with | NR2C2 |
| HMMR | interacts with | ZFX |
| HMMR | interacts with | GTF2E2 |
| HMMR | interacts with | FOXA3 |
| HMMR | interacts with | MEF2D |
| HMMR | interacts with | GABPA |
| HMMR | interacts with | TRIM22 |
| HMMR | interacts with | SP2 |
| HMMR | interacts with | PBX2 |
| HMMR | interacts with | ZNF589 |
| HMMR | interacts with | SMARCA4 |
| HMMR | interacts with | SIRT6 |
| HMMR | interacts with | SAP30 |
| HMMR | interacts with | NCOR1 |
| HMMR | interacts with | IRF1 |
| HMMR | interacts with | DDX20 |
| HMMR | interacts with | CREB1 |
| HMMR | interacts with | ZNF644 |
| HMMR | interacts with | ATF1 |
| HMMR | interacts with | NR2F6 |
| HMMR | interacts with | NFIL3 |
| HMMR | interacts with | KDM5A |
| HMMR | interacts with | ETV4 |
| HMMR | interacts with | SMAD5 |
| HMMR | interacts with | KLF16 |
| HMMR | interacts with | TAF7 |
| FN1 | interacts with | CREB1 |
| FN1 | interacts with | ARID4B |
| FN1 | interacts with | ATF4 |
| FN1 | interacts with | ZFP2 |
| FN1 | interacts with | ATF2 |
| FN1 | interacts with | ZNF644 |
| FN1 | interacts with | SSRP1 |
| FN1 | interacts with | BATF |
| FN1 | interacts with | RERE |
| FN1 | interacts with | PPARG |
| FN1 | interacts with | RARA |
| FN1 | interacts with | ZNF384 |
| FN1 | interacts with | HMG20B |
| FN1 | interacts with | ATF1 |
| FN1 | interacts with | GATAD1 |
| FN1 | interacts with | ZNF580 |
| FN1 | interacts with | TFAP4 |
| FN1 | interacts with | GATAD2A |
| FN1 | interacts with | HHEX |
| FN1 | interacts with | SOX13 |
| FN1 | interacts with | TGIF2 |
| FN1 | interacts with | FOSL1 |
| FN1 | interacts with | CEBPG |
| FN1 | interacts with | TEAD1 |
| FN1 | interacts with | KLF11 |
| FN1 | interacts with | ZNF324 |
| FN1 | interacts with | DMAP1 |
| FN1 | interacts with | MLX |
| FN1 | interacts with | ZNF197 |
| FN1 | interacts with | NR2F6 |
| FN1 | interacts with | ZHX2 |
| FN1 | interacts with | NFYC |
| FN1 | interacts with | RXRB |
| FN1 | interacts with | TEAD3 |
| FN1 | interacts with | NFIL3 |
| FN1 | interacts with | DRAP1 |
| FN1 | interacts with | MBD1 |
| FN1 | interacts with | BCL6 |
| FN1 | interacts with | MLLT1 |
| FN1 | interacts with | ETV4 |
| FN1 | interacts with | ZBTB26 |
| FN1 | interacts with | POLR2H |
| FN1 | interacts with | KLF16 |
| FN1 | interacts with | KLF9 |
| FN1 | interacts with | GATA4 |
| CCNB1 | interacts with | KDM5A |
| CCNB1 | interacts with | ZNF175 |
| CCNB1 | interacts with | POLR2A |
| CCNB1 | interacts with | ETV4 |
| CCNB1 | interacts with | ZBTB26 |
| CCNB1 | interacts with | ZNF121 |
| CCNB1 | interacts with | WT1 |
| CCNB1 | interacts with | ZNF263 |
| CCNB1 | interacts with | MAX |
| CCNB1 | interacts with | SMAD5 |
| CCNB1 | interacts with | TAF7 |
| CCNA2 | interacts with | POLR2H |
| CCNA2 | interacts with | NFE2 |
| CCNA2 | interacts with | KLF16 |
| CCNA2 | interacts with | ZBTB40 |
| CCNA2 | interacts with | ELF3 |
| CCNA2 | interacts with | TAF7 |
| CCNA2 | interacts with | ELF1 |
| CCNA2 | interacts with | KLF9 |
| CCNA2 | interacts with | GATA2 |
| CCNA2 | interacts with | GATA4 |
| CCNA2 | interacts with | RFXANK |
| CCNA2 | interacts with | ZNF71 |
| CCNA2 | interacts with | ZBTB11 |
| CCNA2 | interacts with | HMBOX1 |
